# The G-quadruplex experimental drug QN-302 impairs liposarcoma cell growth by inhibiting *MDM2* expression and restoring p53 levels

**DOI:** 10.1093/nar/gkaf085

**Published:** 2025-02-13

**Authors:** Beatrice Tosoni, Eisa Naghshineh, Irene Zanin, Irene Gallina, Lorenzo Di Pietro, Loredana Cleris, Matteo Nadai, Mara Lecchi, Paolo Verderio, Pietro Pratesi, Sandro Pasquali, Nadia Zaffaroni, Stephen Neidle, Marco Folini, Sara N Richter

**Affiliations:** Department of Molecular Medicine, University of Padua, Via A. Gabelli, 63, 35121 Padua, Italy; Molecular Pharmacology Unit, Department of Experimental Oncology, Fondazione IRCCS Istituto Nazionale dei Tumori di Milano, Via G. A. Amadeo, 42, 20133 Milan, Italy; Department of Molecular Medicine, University of Padua, Via A. Gabelli, 63, 35121 Padua, Italy; Department of Molecular Medicine, University of Padua, Via A. Gabelli, 63, 35121 Padua, Italy; Molecular Pharmacology Unit, Department of Experimental Oncology, Fondazione IRCCS Istituto Nazionale dei Tumori di Milano, Via G. A. Amadeo, 42, 20133 Milan, Italy; Molecular Pharmacology Unit, Department of Experimental Oncology, Fondazione IRCCS Istituto Nazionale dei Tumori di Milano, Via G. A. Amadeo, 42, 20133 Milan, Italy; Department of Molecular Medicine, University of Padua, Via A. Gabelli, 63, 35121 Padua, Italy; Bioinformatic and Biostatistics Unit, Department of Epidemiology and Data Science, Fondazione IRCCS Istituto Nazionale dei Tumori di Milano, Via G. Venezian, 1, 20133 Milan, Italy; Bioinformatic and Biostatistics Unit, Department of Epidemiology and Data Science, Fondazione IRCCS Istituto Nazionale dei Tumori di Milano, Via G. Venezian, 1, 20133 Milan, Italy; Bioinformatic and Biostatistics Unit, Department of Epidemiology and Data Science, Fondazione IRCCS Istituto Nazionale dei Tumori di Milano, Via G. Venezian, 1, 20133 Milan, Italy; Molecular Pharmacology Unit, Department of Experimental Oncology, Fondazione IRCCS Istituto Nazionale dei Tumori di Milano, Via G. A. Amadeo, 42, 20133 Milan, Italy; Molecular Pharmacology Unit, Department of Experimental Oncology, Fondazione IRCCS Istituto Nazionale dei Tumori di Milano, Via G. A. Amadeo, 42, 20133 Milan, Italy; School of Pharmacy, University College London, London WC2N 1AX, United Kingdom; Molecular Pharmacology Unit, Department of Experimental Oncology, Fondazione IRCCS Istituto Nazionale dei Tumori di Milano, Via G. A. Amadeo, 42, 20133 Milan, Italy; Department of Molecular Medicine, University of Padua, Via A. Gabelli, 63, 35121 Padua, Italy; Microbiology and Virology Unit, Padua University Hospital, 35128 Padua, Italy

## Abstract

Well-differentiated/dedifferentiated liposarcomas (WD/DDLPSs) account for ∼60% of all liposarcomas. They have a poor prognosis due to limited therapeutic options. WD/DDLPSs are characterized by aberrant expression of mouse double minute 2 (*MDM2*), which forms G-quadruplexes (G4s) in its promoter. Here, we investigated the possibility of targeting WD/DDLPSs with small molecules against the *MDM2* G4s. Among the molecules tested, the naphthalene diimide derivative QN-302 significantly impaired WD/DDLPS cell growth and its activity strikingly paralleled cell-specific G4 abundance as measured by CUT&Tag and RNA sequencing analysis. QN-302 stabilized *MDM2* G4s at the P2 inducible promoter and prevented polymerase progression from the constitutive P1 promoter, thereby inhibiting the formation of full-length *MDM2* transcripts. This resulted in the accumulation of p53 through the p53-MDM2 autoregulatory feedback loop, ultimately leading to apoptotic cell death. In patient-derived xenograft mouse models, QN-302 treatment reduced tumour volume distribution and was well tolerated. We have identified a novel and effective therapeutic strategy to reduce *MDM2* expression and promote p53 reactivation in tumours harbouring wild-type TP53, such as WD/DDLPSs.

## Graphical Abstract

**Graphical Abstract:**
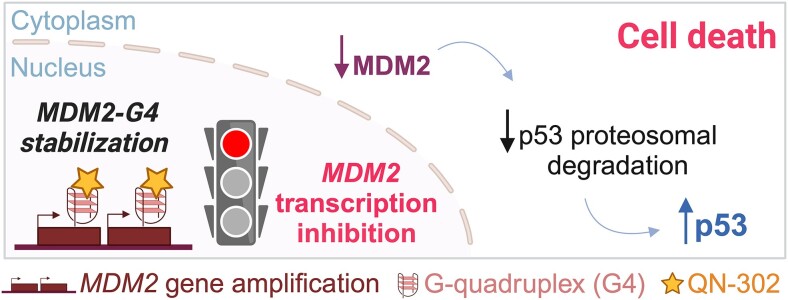

## Introduction

Liposarcoma (LPS) is the most common soft-tissue sarcoma subtype [1], accounting for about 20% of all mesenchymal cancers [2, 3]. Well-differentiated liposarcoma (WDLPS) and dedifferentiated liposarcoma (DDLPS) account for 60% of all LPSs [4]. While WDLPS is a locally aggressive tumour accounting for rare cases of metastasis, DDLPS, which may develop from the transition of WDLPS to a non-lipogenic aggressive sarcoma or can arise *de novo*, is indolent and characterized by higher rates of local recurrence and distant metastasis [4]. At the molecular level, both WDLPS and DDLPS share common genetic features, such as the presence of supernumerary rings or giant rod chromosomes arising as the consequence of alterations occurring within the 12q13-15 gene region, leading to oncogene amplification, notably for the two genes mouse double minute 2 (*MDM2*) and cyclin-dependent kinase 4 (*CDK4*) [4]. DDLPS harbours further genomic alterations, including the amplification of 1p32 and 6q23 genomic regions housing genes involved in the dedifferentiation process [2]. Surgery is the mainstay treatment for localized disease, while preoperative radiotherapy has been reported to reduce local recurrence only in less aggressive DDLPS (malignancy grade 2) and in WDLPS [5]. Conversely, DDLPS patients with multiple recurrences or advanced unresectable or metastatic disease are treated with first-line doxorubicin or doxorubicin-based regimens [6], while ifosfamide, trabectedin, eribulin, dacarbazine, and pazopanib represent further treatment options [4]. Recently, molecular-targeted therapies and immunotherapy have emerged as potential therapeutic options for patients with advanced or metastatic disease [7]. However, the dismal prognosis and the low objective response rate to currently available therapeutic approaches, particularly for DDLPS, underlie the urgent need for improving the knowledge of the molecular characteristics of the disease with the aim of identifying and validating specific actionable targets to inform the design of novel biology-driven therapies [6].

*MDM2* amplification is a major driver gene in both WDLPS and DDLPS with diagnostic and therapeutic relevance [7]. The oncogenic properties of MDM2 stem from its ability to act as one of the main negative regulators of the tumour suppressor p53 protein [7]. *MDM2* gene transcription is primarily controlled by two distinct promoters [8]. The P1 promoter, located upstream of exon 1, is responsible for the constitutive expression of the *MDM2* gene [9]. The P2 promoter, located within the first intron, is induced by several transcription factors, most notably p53, in response to different stimuli [8, 9]. Specifically, p53 binds to the responsive elements (REs; p53-REs) within the P2 promoter and enhances the expression of the MDM2 protein. In turn, MDM2 binds and masks the transactivation domain of p53 and targets p53 for proteasomal degradation [8, 10]. This mechanism, referred to as the p53-MDM2 feedback loop, is necessary to maintain homeostatic levels of p53 in normal unstressed cells. As a consequence, *MDM2* gene amplification/overexpression acts as an oncogenic event by diminishing the tumour-suppressive function of p53 in tumours bearing the wild-type form of the protein [10].

G-quadruplexes (G4s) are non-canonical nucleic acid four-stranded secondary structures that can form in G-rich sequences, typically containing up to four short G-tracts. G4s result from the stacking of two or more planar associations of four guanines (G-quartets) [11]. G-quartets are held together by Hoogsteen-type hydrogen bonds in a square co-planar arrangement coordinated with a central monovalent cation (preferably K^+^ and Na^+^) [11]. The human genome contains thousands of G4-forming sequences [12]. It has been reported that G4s form in nucleosome-depleted chromatin regions at gene promoters, around the transcription start sites (TSSs) of highly transcribed genes [11, 13–16]. This evidence alongside the observation that many tumour-associated genes harbour G4-forming sequences within their promoters [17] suggests a pivotal role of G4s in gene expression regulation in cancer. Many small molecule compounds have been indeed characterized as G4 ligands and some have displayed promising anti-cancer effects in different tumour experimental models [17]. Among these compounds, QN-302 has advanced beyond the experimental and pre-clinical evaluation stage and is currently being evaluated in a phase I clinical trial for the treatment of advanced solid tumours [18].

These observations have provided the rationale to explore G4 targeting as a therapeutic approach in cancer [17]. In this regard, we recently identified and characterized a G4-forming region within the P2 promoter of *MDM2* [19], thus identifying the *MDM2* oncogene as a possible therapeutic target amenable to G4 stabilization in LPS. In this study, a therapeutic approach based on G4 stabilization aimed at interfering with *MDM2* expression in WDLPS and patient-derived DDLPS cell lines was pursued. We focused on QN-302 [18, 20, 21], which belongs to the most promising family of G4 ligands characterized by a naphthalene diimide (NDI) core and is able to bind to G4 structures by stacking on the outer G-quartets [22]. We report here that QN-302 shows excellent inhibitory activity on the growth of human WD/DDLPS cell lines compared with normal human subcutaneous pre-adipocytes (PAs). By deciphering the mechanism of action of QN-302, we show that binding of QN-302 to the G4-positive P2 promoter region inhibits *MDM2* transcription, leading to a pronounced increase in p53 protein levels and the consequent inhibition of cell growth and induction of apoptosis *in vitro* and tumour volume (TV) reduction *in vivo*. This work thus highlights an innovative potential therapeutic strategy for WD/DDLPS.

## Materials and methods

### Cell cultures, drugs, and reagents

The human WDLPS cell line 93T449 (CRL-3043; ATCC, Rockville, MD, USA), named WD in the text, was cultured in the Roswell Park Memorial Institute-1640 (RPMI-1640) medium (Thermo Fisher Scientific Inc., Waltham, MA, USA) supplemented with 10% fetal bovine serum (Thermo Fisher) and 1% penicillin–streptomycin (Merck KGaA, Darmstadt, Germany) solution. Patient-derived DDLPS cell lines LS-GD-1 and LS-BZ-1, named DD-1 and DD-2, respectively in the text, were originated as previously described [23], and cultured in Dulbecco’s modified Eagle’s medium/nutrient mixture F-12 supplemented with 10% fetal bovine serum and 1% penicillin–streptomycin (Merck). Normal human subcutaneous PAs (PT-5020; Lonza Milan Srl, Treviglio, Italy), were cultured in Preadipocyte Basal Medium 2 (Lonza) supplemented with gentamicin, 7.5 mM l-glutamine, and 2% fetal bovine serum. The p53-null PC-3 (CRL-1435) prostate cancer cell line was obtained from ATCC and maintained as a monolayer in the logarithmic growth phase in RPMI-1640 medium (Lonza) supplemented with 10% fetal calf serum at 37°C in a humidified incubator at 5% CO_2_.

The NDI derivatives QN-302 and CM03 had been synthesized (>98% purity) as previously described [20, 24]. Pyridostatin (PDS) and PhenDC3 were purchased from Merck. All compounds were dissolved in dimethyl sulfoxide and stored as a stock solution at −20°C. Working solutions were prepared in a complete cell culture medium just before use.

### Cell viability assay

G4 ligand (PDS, PhenDC3, QN-302, and CM03) cell growth inhibition activity was assessed by MTT Cell Growth Kit (Merck), according to the manufacturer’s instructions. Briefly, PA, WD, DD-1, and DD-2 cells were plated in 96-well plates and incubated overnight at 37°C in a humidified atmosphere at 5% CO_2_. Cells were seeded at 75%–80% confluence, according to their doubling time. Cells were then exposed for 24 h to serial dilutions of each compound, as indicated in the results section. At the end of treatment, 5 mg/ml 3-(4,5-dimethylthiazol-2-yl)-2,5-diphenyltetrazolium bromide solution was added to each well. After 4 h incubation at 37°C and the solubilization of formazan with isopropanol/0.04 N HCl, the absorbance was measured at 620-nm wavelength using the Varioskan LUX Multimode Microplate Reader (Thermo Fisher). The cell growth inhibitory activity of tested compounds was reported as the percentage of viable cells with respect to untreated cells (solvent) and the IC_50_ (i.e. the concentration of compound required to inhibit cell viability by 50%) were calculated using the function Log (inhibitor) versus normalized response in GraphPad Prism version 10.0.3.

### Cleavage under targets and tagmentation

Cleavage under targets and tagmentation (CUT&Tag) experiments were performed on fresh WDLPS, DDLPS, and PA cells (100 000 cells/sample), with or without 0.8 μM QN-302 for 4 h, as previously reported [25]. Briefly, once collected and washed with wash buffer, cells were immobilized on concanavalin A-coated magnetic beads (Bangs Laboratories Inc., Fishers, IN, USA) and incubated overnight at 4°C with the FLAG-tagged anti-G4 BG4 antibody (500 ng). The BG4 antibody was expressed, purified, and validated as previously described [26]. Samples were then incubated for 1 h at room temperature (RT) first with anti-FLAG antibody (1:100 dilution) (Merck), followed by rabbit anti-mouse IgG (Merck). Bead-bound cells were then washed in dig-wash buffer and subjected to binding with pA–Tn5 adapter complex, followed by tagmentation at 37°C for 1 h. Once tagmentation was stopped, libraries were purified by phenol–chloroform extraction and subjected to amplification using NEBNext High-Fidelity 2× PCR Master Mix with uniquely barcoded i5 and i7 primers. CUT&Tag libraries were then size selected using Agencourt AMPure XP Beads (Beckman Coulter, Pasadena, CA, USA) and size distribution was analysed with Bioanalyzer (Agilent Technologies, Milan, Italy). Samples were sequenced on an Illumina NextSeq500 platform using 38-bp reads and paired-end sequencing methods.

### CUT&Tag data analysis

CUT&Tag data were processed as previously reported [25] with minor modifications. Sequencing data were analysed with the open-source web platform Galaxy (www.usegalaxy.org) together with R (version 4.2.1 – RStudio, version 2022.07.0). Reads were aligned to the human reference genome (GRCh38) using Bowtie 2 (version 2.5.0) [27] after FastQC quality control (http://www.bioinformatics.babraham.ac.uk/projects/fastqc). G4 peaks were called with MACS2 (version 2.2.7.1) [28] (-gsize 2.7e9 -bw 150 -qvalue 0.05) and high-confidence peaks were defined as those that were consistently detected in at least two out of three independent biological replicates using the BEDTools [29] intersect command. Peaks annotation was performed with ChIPseeker (version 1.28.3) [30]. Reads Per Genome Coverage (RPGC)-normalized tracks were generated with deepTools (version 3.5.1) [31] bamCoverage and merged with bigwigCompare function after scaling with respect to the sample with the lowest sequencing depth. Sample profiles were plotted using the R package ‘Gviz’ [32]. Bar and donut plots were generated using GraphPad Prism version 10.0.3.

### RNA-seq analysis

Total RNA was extracted from WDLPS, DDLPS, and PA cells (2.0 × 10^5^) treated with the indicated amounts of QN-302 using GeneJET RNA Purification Kit (Thermo Fisher) according to the manufacturer’s instructions. Three independent biological replicates were analysed and sequenced single-end on Illumina NextSeq500 platform using 75-bp reads. Library construction and bioinformatic analysis were performed as previously reported [33]. Briefly, after sequencing adapter exclusion using Trimmomatic (0.38.1) [34], reads were aligned to the human reference genome (GRCh38) using HISAT2 (version 2.2.1) [35]. Genes differentially expressed after QN-302 treatment (upregulated: Log_2_FC > 0.5 and *P*-value < .01; downregulated: Log_2_FC < −0.5 and *P*-value < .01) were identified with Bioconductor package DESeq2 [36]. We used the Bioconductor package ‘goseq’ [37] to perform the Kyoto Encyclopedia of Genes and Genomes (KEGG) signaling pathway enrichment analysis on differentially expressed genes (DEGs) for QN-302-treated versus untreated condition that were analysed by DESeq2 [36]. The Bioconductor package ‘pathview’ [38] was used to visualize enriched signaling pathways and show the upregulated (Log_2_FC > 0.5 and *P*_adj_ < .05) and downregulated (Log_2_FC < −0.5 and *P*_adj_ < .05) genes in the pathway.

### Circular dichroism spectroscopy

The capacity of QN-302 to stabilize the MDM2-G4 (5′-TGGGGGCTCGGGGCGCGGGGCGCGGGGCATGGGGC-3′) [19] was determined by circular dichroism (CD) spectroscopy. The MDM2-G4 oligonucleotide (2 μM) was diluted in lithium cacodylate buffer (10 mM; pH 7.4) with or without 25 mM KCl. After heat-denaturation at 95°C for 5 min, samples were kept at RT overnight. For the experiments in the presence of QN-302, the compound was added 4 h after oligonucleotide denaturation at final concentration of 8 μM. CD spectra were recorded on a Chirascan-Plus (Applied Photophysics, Leatherhead, UK) equipped with a Peltier temperature controller using a quartz cell of 5-mm optical path length. To determine the melting temperature (*T*_m_), spectra were acquired over a temperature range of 20°C–90°C, with temperature increase of 5°C/min over a wavelength range of 230–320 nm. *T*_m_ values were calculated according to the van’t Hoff equation, applied for a two-state transition from a folded to unfolded state. Once CD spectra were baseline corrected for signal contribution of the buffer, the ellipticities were converted to mean residue ellipticity (θ) = deg × cm^2^ × dmol^−1^ (molar ellipticity).

### Chromatin immunoprecipitation assay for p53

A chromatin immunoprecipitation (ChIP) assay targeting p53 was conducted using 5 million WD cells, either untreated or treated with the indicated concentrations of QN-302 for 4 h. The cells were cross-linked using 1% formaldehyde (Thermo Fisher) for 10 min at RT, and the reaction was quenched by adding glycine to a final concentration of 125 mM. Cross-linked cells were resuspended in nuclei isolation buffer 1 [50 mM HEPES–NaOH (pH 7.5), 140 mM NaCl, 1 mM ethylenediaminetetraacetic acid (EDTA), 10% glycerol, 0.5% IGEPAL, 0.25% Triton X-100, and 1× Protease Inhibitor] and incubated for 15 min at 4°C. This was followed by a second lysis step in nuclei isolation buffer 2 [10 mM Tris–HCl (pH 8.0), 200 mM NaCl, 1 mM EDTA, 0.5 mM egtazic acid (EGTA), and 1× Protease Inhibitor] with an additional 15-min incubation at 4°C. The isolated nuclei were then resuspended in sonication buffer [10 mM Tris–HCl (pH 8.0), 100 mM NaCl, 1 mM EDTA, 0.5 mM EGTA, 0.1% sodium deoxycholate, 0.5% sodium lauroyl sarcosinate, and 1× Protease Inhibitor], and the chromatin was sheared to an average size of 200–700 bp using a Bioruptor (Diagenode SA, Belgium, Europe) for 15 cycles (30 s ON/30 s OFF). Pre-cleared, sonicated chromatin was subjected to immunoprecipitation using an anti-p53 antibody (Cell Signaling) or a rabbit anti-mouse IgG antibody (Cell Signaling) in IP buffer [100 mM Tris–HCl (pH 8.0), 100 mM NaCl, 5 mM EDTA, 0.3% sodium dodecyl sulfate (SDS), and 1.7% Triton X-100] overnight at 4°C. The following day, the immunocomplexes were incubated with a 1:1 mix of protein A and G Dynabeads (Thermo Fisher) for 4 h at 4°C and subjected to sequential washes using four different buffers (each applied twice): wash buffer 1 [20 mM Tris–HCl (pH 8.0), 150 mM NaCl, 5 mM EDTA, 1% Triton X-100, 0.2% SDS, and 5% sucrose], wash buffer 2 [50 mM HEPES–NaOH (pH 7.5), 500 mM NaCl, 1 mM EDTA, 0.1% sodium deoxycholate, and Triton X-100], wash buffer 3 [10 mM Tris–HCl (pH 8.0), 250 mM LiCl, 1 mM EDTA, 0.5% sodium deoxycholate, and 0.5% IGEPAL], and wash buffer 4 [10 mM Tris–HCl (pH 8.0) and 1 mM EDTA]. The immunocomplexes bound to Dynabeads were eluted in elution buffer [10 mM Tris–HCl (pH 8.0), 1 mM EDTA, and 1% SDS], and cross-links were reversed at 65°C for 4 h. The samples were subsequently treated with RNase A for 30 min at 37°C and proteinase K (0.2 μg/ml) for 1 h at 55°C. DNA was purified using the MinElute PCR Purification Kit (Qiagen, Hilden, Germany), following the manufacturer’s instructions. Quantitative polymerase chain reaction (qPCR) was performed using SYBR™ Green PCR Master Mix (Thermo Fisher) in QuantStudio™ 3 Real-Time PCR System (Thermo Fisher) to measure the relative amounts of ChIP DNA relative to inputs. The following primer pairs were utilized for qPCR: (i) *MDM2* primer pairs (forward: 5′-TTCAGTGGGCAGGTTGACTC-3′; reverse: 5′-GGTGCTTACCTGGATCAGCA-3′) targeted the p53-RE within the *MDM2* P2 promoter region; (ii) positive-region primer pairs (forward: 5′-TTTGGGCGTGGAGATAAGGTGGA-3′; reverse: 5′-GGGCGTGTGTGTGTGTGTGT-3′) specific to the p53-RE within the *CDKN1A* gene [39] were used as a positive control; (iii) negative-region primer pairs (forward: 5′-ACGACAAAGAAAACGCCACA-3′; reverse: 5′-GATTCGATGGCGTCCCTGTA-3′) amplified a region located ∼20 kb downstream of the *MDM2* P2 promoter that does not contain p53-binding sites. The primers were purchased from Sigma–Aldrich (Merck).

### Cell-based experiments

To assess the effect of QN-302 on DDLPS on the expression of target genes over time, cells were seeded at the appropriate density in 25-cm^2^ tissue flasks and incubated at 37°C in a humidified atmosphere at 5% CO_2_. The day after, cells were treated with the indicated amounts of the compounds for the indicated timepoints, according to the experimental design. Cells were then collected and subsequently analysed. To assess cell growth in the long term, DD-1 and DD-2 cells were seeded at appropriate density in 25-cm^2^ tissue culture flasks, exposed to 0.4 μM of freshly dissolved QN-302. Cells were then collected and counted in a particle counter (Coulter Counter, Coulter Electronics, Luton, UK) after 24, 48, and 72 h of drug exposure.

For the transfection procedure, cells seeded at the appropriate density and allowed to attach for 24 h at 37°C in a humidified atmosphere at 5% CO_2_ were incubated with Lipofectamine-2000^TM^ (Thermo Fisher) in Opti-MEM I (Thermo Fisher). After 15 min at RT, lipid-treated cells were added with Opti-MEM I medium containing 50 nM of a small interfering RNA (siRNA) directed against p53 (sip53) (sc-29735; Santa Cruz Biotechnology Inc., Dallas, TX, USA) or a control siRNA (siCTR) (sc-3700; Santa Cruz Biotechnology), incubated for 4 h at 37°C and subsequently exposed over time to a subtoxic amount of QN-302. Cells were then harvested according to the timeline of each experiment and subsequently analysed.

### Quantitative reverse transcription PCR

Cells were seeded in 35-mm dish and treated with the indicated amounts of QN-302. Total RNA was extracted using GeneJET RNA Purification Kit (Thermo Fisher) according to the manufacturer’s instructions and subjected to DNase digestion with TURBO DNA-free Kit (Thermo Fisher). Total RNA (250 ng) was reverse transcribed by TaqMan™ Reverse Transcription Reagents kit according to the manufacturer’s instructions and using the oligo (dT_16_) to specifically detect the total messenger RNA (mRNA). The complementary DNA (cDNA) was amplified using SYBR™ Green PCR Master Mix (Thermo Fisher) in the presence of gene-specific primer pairs: full-length *MDM2* 5′-ACGACAAAGAAAACGCCACA-3′ (forward) and 5′-GATTCGATGGCGTCCCTGTA-3′ (reverse); *MDM2* P1-derived transcripts 5′-GGAAACTGGGGAGTCTTGAGGGAC-3′ (forward) and 5′-TCCGAAGCTGGAATCTGTGAGGTG-3′ (reverse) [9]; *MDM2* P2-derived transcripts 5′-GATTGGAGGGTAGACCTGTGGGCA-3′ (forward) and 5′-TCCGAAGCTGGAATCTGTGAGGTG-3′ (reverse) [9]; TP53 5′- TCTACTGGGACGGAACAGCT-3′ (forward) and 5′-GGTGAGGCTCCCCTTTCTTG-3′ (reverse); and ACTB 5′-TCACTGAGCGCGGCTACA-3′ (forward) and 5′-CCTTAATGTCACGCACGATTTC-3′ (reverse). All the primer pairs were purchased from Merck. The amplification reactions were run on QuantStudio™ 3 Real-Time PCR System (Thermo Fisher) under the following conditions: one cycle at 95 °C for 10 min and then 15 s at 95 °C, 30 s at 60 °C and 30 s at 72 °C for 40 cycles. Data were analysed using the 2^−ΔΔCt^ method [39] and changes in the levels of mRNA were expressed as relative quantity in treated versus untreated cells (calibrator sample) using β-actin as normalizer.

To capture nascent transcripts, Click-iT Nascent RNA Capture Kit (#10365, Thermo Fisher) was used according to the manufacturer’s instructions. Briefly, 1 × 10^6^ PC-3 cells were plated in 25-cm^2^ tissue culture flasks and incubated for 24 h at 37°C in a humidified atmosphere at 5% CO_2_. After 2- and 8-h exposure to QN-302 (0.8 μM), both untreated and treated cells were pulse-labelled with 5-ethynyl uridine (EU) at 0.2 mM final concentration and incubated in the absence or presence of QN-302 for additional 2 h. EU-labelled nascent RNA was then captured from total RNA (2.5 μg) by a copper-catalysed click reaction with 0.5 mM biotin azide and pulled down by Dynabeads™ MyOne™ Streptavidin T1 beads. After 3× washes, nascent RNA was directly used as a template for cDNA synthesis using the SuperScript™ VILO™ cDNA Synthesis Kit (#11754050, Thermo Fisher) following the optimized protocol according to Click-iT Nascent RNA Capture Kit and subsequently amplified by qPCR using P1 and P2 specific primer pairs as described above. Data have been reported as 2^−ΔCt^ using β-actin as normalizer.

### Western blotting and antibody arrays

Cells were seeded in a 60-mm dish at a density of 4.0 × 10^5^, grown overnight, and then treated with QN-302 (0.4, 0.6, and 0.8 μM). Cells were lysed in RIPA buffer [150 mM NaCl, 1% IGEPAL, 0.1% SDS (pH 7.5), 50 mM Tris–HCl, and 0.5% sodium deoxycholate] and protein concentration was quantified using the Pierce™ BCA Protein Assay Kit (Thermo Fisher). Proteins (20 μg) were fractionated by sodium dodecyl sulfate–polyacrylamide gel electrophoresis and transferred onto nitrocellulose (0.22 μm) filters (AmershamTM Protan, GE Healthcare Life Sciences, Milan, Italy). The filters were blocked in Tris-buffered saline (TBS) and 5% skim milk and incubated overnight at 4°C with the following primary antibodies: anti-MDM2 (#86934; Cell Signaling Technology Inc., Danvers, USA), anti-PARP1 (cleaved Asp214, Asp215) (44-698G; Thermo Fisher), anti-Caspase 3 (cleaved Asp175) (#9661; Cell Signaling Technology), anti-Caspase 3 (procaspase-3) (sc-7272; Santa Cruz Biotechnology), anti-γH2A.X (Ser139) (05-636; Merck), anti-p53 (sc-126; Santa Cruz Biotechnology), anti-ATRX (ab97508; Abcam, Cambridge, UK), and anti-TOP3A (sc-11257; Santa Cruz Biotechnology). Anti-α-tubulin (T5168; Merck), anti-GAPDH (G8795; Merck), and anti-vinculin (V9131; Merck) were used as housekeeping genes. Membranes were then probed with horseradish peroxidase-conjugated goat anti-mouse IgG (AP308P; Merck), goat anti-rabbit IgG (12-348; Merck), or donkey anti-goat (ab6885; Abcam) secondary antibodies and images captured by Alliance Uvitec (Uvitec Ltd. Cambridge, Cambridge, UK).

To evaluate the phosphorylation status of p53 at multiple serine residues (S15, S392, and S46), a multiplex antibody array (Proteome Profiler Human Phospho-Kinase Array Kit, #ARY003C; R&D Systems, Bio-Techne SRL, Milan, Italy) was used, according to the manufacturer’s instructions. Briefly, proteins (400 μg) from untreated and QN-302-treated (IC_50_ at 48 h) DDLPS cells were incubated with membranes containing spotted antibodies in duplicate to bind to specific target proteins present in the sample. Dot blots were visualized using Pierce ECL Western Blotting Substrate (Thermo Scientific™) and images were captured by Alliance Uvitec (Uvitec Ltd. Cambridge).

### Annexin V staining

The occurrence of apoptotic cell death in cells exposed to QN-302 was assessed by eBioscience™ Annexin V Apoptosis Detection Kit (Thermo Fisher), according to the manufacturer’s instructions. Briefly, cells were trypsinized, washed, and resuspended in 1× binding buffer. Cell suspensions (100 μl) were then stained with Annexin V–fluorescein isothiocyanate conjugate followed by flow cytometric analysis with the FACS Cytofluorometer BD LSR II instrument (BD Biosciences, NJ, USA). The combined use of propidium iodide necessary to distinguish between early and late apoptotic cells was excluded to avoid analysis interference due to the fluorescence emission at the same wavelength of QN-302. Data were analysed with BD FACSDiva™ Software and reported as per cent of positive-Annexin V within the same and appropriate gate.

### *In vivo* studies

Tumour fragments of the established DDLPS PDX-BZ-1 model [23], that is cognate to DD-2 cell line, were grafted subcutaneously into the left flank of 6-week-old female NOD.CB17-*Prkdc^scid^*/NCrCrl (SCID) mice (Charles River Laboratories, Calco, Italy). Mice were maintained in a pathogen-free facility where temperature and humidity were kept constant and had free access to food and water. For each experimental block (*b* = 2), mice were randomized into 12 cages each housing two mice and divided into four groups. The treatment dose and schedule for each drug were selected from the literature. Briefly, after dilution in physiological solution, doxorubicin (Adriblastina, Pfizer Italia) was administered at 2.5 mg/kg intravenously (i.v.) every 7 days for three times (q7d × 3) [23]. QN-302 (1 mg/kg) and CM03 (10 mg/kg) were prepared as previously described and administered i.v. twice a week for 5 times (q3-4d × 5) [20, 40]. Tumour growth was monitored by biweekly measurement of tumour diameters with a Vernier calliper. TV was calculated according to the formula: *TV* (mm^3^) = *d*^2^ × *D*/2, where *d* and *D* are the shortest and the longest diameters, respectively. Drug treatment activity was reported as Log_10_-transformed TV at the end of treatment (day 16). Treatment toxicity was evaluated by body weight loss and occurrence of lethal toxicity. Tumour specimens were collected at the end of the experiments, snap-frozen, and subsequently analysed.

A three-way analysis of variance (ANOVA) was carried out to investigate the effects of treatment on TV (Log_10_-transformed), by adjusting for the experimental block variable and initial animal body weight.

*In vivo* experiments were authorized by the Italian Ministry of Health according to the national law (project approval code 3/2023-PR) and in compliance with international policies and guidelines.

### Statistical analysis

Statistical analysis was performed using GraphPad Prism software (GraphPad Prism 10.0.3). The data have been reported as mean values with relative dispersion and precision measures (e.g. mean ± SEM) from at least three independent experiments. For multiple comparisons between groups, significance was tested with two-way ANOVA (Tukey’s) test. Non-parametric Mann–Whitney and Wilcoxon tests were used to analyse difference between samples. The proper statistical test applied for each experiment is reported in the corresponding figure legend. *P*-values < .05 were considered statistically significant.

## Results

### QN-302 remarkably impairs the viability of LPS cells

**Figure 1.**
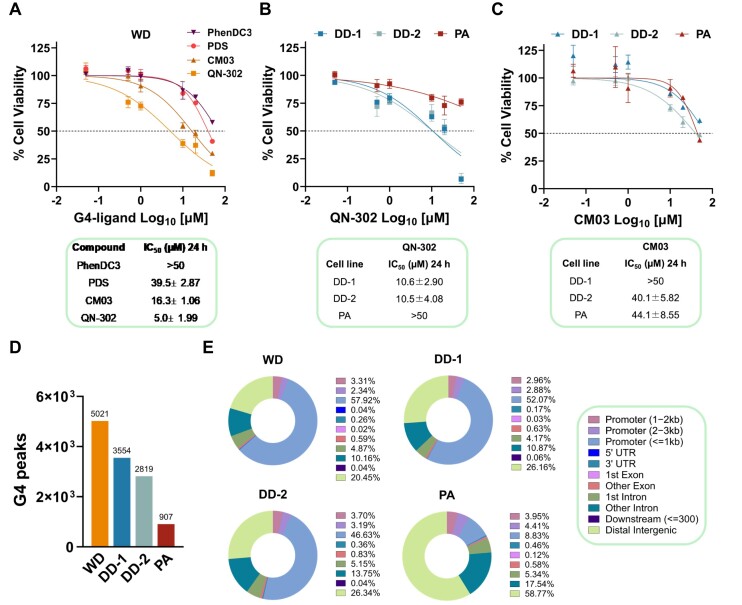
The potent cell growth inhibitory activity of QN-302 is linked to the specific G4 landscape characteristic of each cell line. (**A**) G4 ligands (PhenDC3, PDS, CM03, and QN-302) cell viability evaluated in WDLPS (WD) cells exposed for 24 h at increasing compound concentrations (0.05, 0.50, 1, 10, 20, and 50 μM). IC_50_ values (i.e. the concentration for each compound required to reduce cell growth by 50%) are reported in the table below: they represent the mean IC_50_ ± SEM of three independent experiments.(**B**) QN-302 and (**C**) CM03 cell viability assessed in DDLPS (DD-1 and DD-2) cells compared with PA cells treated with increasing compound concentrations (0.05, 0.50, 1, 10, 20, and 50 μM) for 24 h. Data are reported as the percentage of cell viability with respect to untreated cells and represent mean values ± SEM of at least three independent experiments. The calculated IC_50_ values are reported in the tables below and represent mean values ± SEM from at least three independent experiments. (**D**) G4 levels in LPS (WD, DD-1, and DD-2) and PA cell lines. The reported G4 CUT&Tag peaks are the high confidence peaks defined as those that were consistently detected in at least two out of three independent biological replicates. (**E**) Donut charts representing the G4 peaks annotation within the genome of WD/DDLPS and PA cells. The percentage of G4 peaks distribution among genomic regions is reported in the legend for each cell line.

We initially evaluated the effect of four G4 ligands known to interfere with the transcription of genes harbouring G4s in their promoters on the viability of LPS cells [17, 18, 20, 21, 24]. In particular, we focused on the ligands PhenDC3 and PDS that are widely used phenanthroline and quinoline derivatives, respectively [17], and the NDI derivatives CM03 and QN-302 that have been recently reported to show significant anti-tumour activity in *in vitro* and *in vivo* models of pancreatic cancer [18, 20, 21, 24, 40]. Specifically, a 24-h exposure of WDLPS (WD) cells to increasing amounts (0.05–50 μM) of the selected compounds resulted in a dose-dependent impairment of cell viability, which was markedly superior for QN-302 and CM03, as indicated by the dose-response curves and the calculated IC_50_ values (Fig. 1A). Based on this evidence, the two NDI derivatives were comparatively tested on two patient-derived DDLPS (DD-1 and DD-2), which represent unique models of treatment-naïve, MDM2-positive, FNCLCC (Fédération Nationale des Centres de Lutte Contre Le Cancer) grade III DDLPS [23], and on normal human PA cells. A 24-h exposure of DDLPS cells to increasing amounts of the compounds resulted in a dose-dependent impairment of cell viability, which was remarkably higher for QN-302 than for CM03 (Fig. 1B and C). QN-302 negligibly affected the viability of PA cells (Fig. 1B), whereas CM03 exerted a dose-dependent inhibition of PA cell viability that was comparable to that observed on DD-1 and DD-2 cells (Fig. 1C). This evidence indicates that a therapeutic window may exist for QN-302, the molecular and biological effects of which were further investigated.

### WD/DDLPS cells are highly enriched in G4s

It has been reported that the available repertoire of cellular G4s and associated transcription factors at gene promoters may be the determinants of cell-specific transcriptional programs [33] and consequently influence the biological activity of G4 ligands. In this regard, we sought to comparatively investigate the G4 landscapes in live LPS and PA cells [12, 25, 41]. CUT&Tag data indicated that the genome-wide distribution of G4 structures in PA cells was much less abundant compared with LPS cells (Fig. 1D). Notably, a trend towards a positive correlation between G4 enrichment and QN-302 cell growth inhibitory activity was observed, as cell lines harbouring the highest number of G4s (i.e. WDLPS > DDLPS, Fig. 1D) showed the greatest sensitivity to the G4 ligand (Fig. 1A and B). Moreover, while G4 distribution in PA primarily coincided with distal intergenic regions (Fig. 1E), the majority of G4s in LPS cells were distributed within gene promoter regions (Fig. 1E).

### QN-302 interferes with *MDM2* expression levels in LPS cells

**Figure 2.**
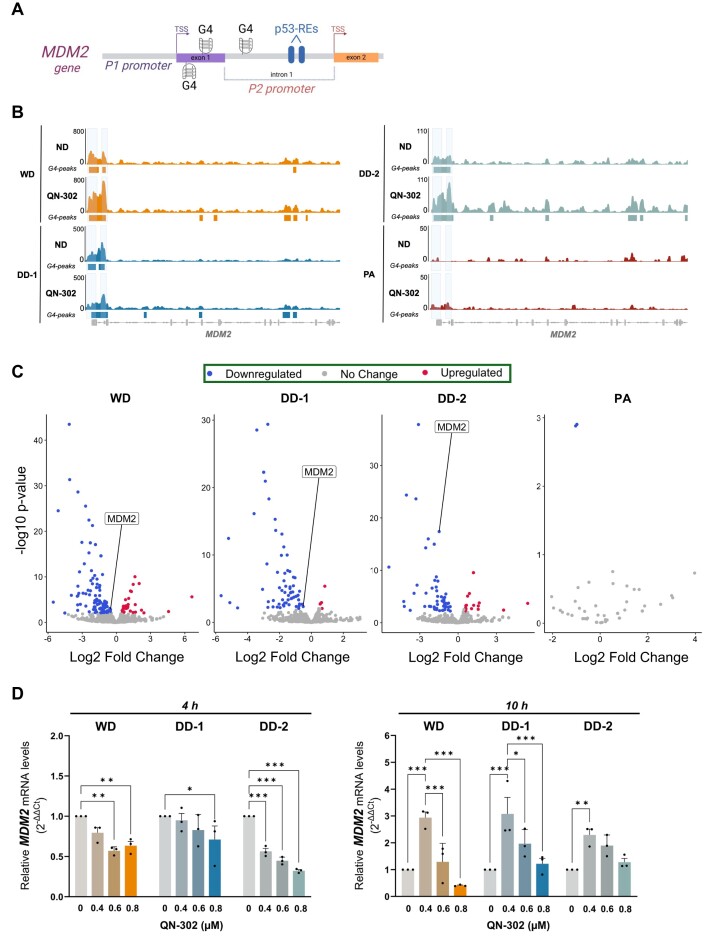
QN-302 effects on *MDM2* expression in WDLPS and DDLPS cell lines. (**A**) Schematic representation of the P1 and P2 promoter regions within the *MDM2* oncogene. The P2 promoter (p53-inducible) is positioned in the first intron between exons 1 and 2; two p53-REs are present in the P2 promoter, downstream of the MDM2-G4 sites. Created in BioRender: Tosoni, B. (2025), https://BioRender.com/l36p271. (**B**) Visualization of G4 CUT&Tag profiles of LPS and PA cells within the *MDM2* genomic region upon treatment with QN-302 (0.8 μM) for 4 h compared with the untreated samples (ND: no drug samples). Reads were aligned to the human genome (hg38) and RPGC-normalized. For each profile, MACS2 called peaks are reported as coloured boxes under each track. Gene annotation is reported at the bottom of the figure. (**C**) Volcano plots representing the differentially expressed G4-holding genes retrieved in WD/DDLPS and PA cell lines by RNA-seq after 4-h exposure to 0.8 μM of QN-302. Only *MDM2*, among all the DEGs (full list reported as supplemental files), is labelled. The genes with -0.5 < Log2FC < 0.5 and P-value > .01 are reported as unchanged expression genes; the genes with Log_2_FC < −0.5 and *P*-value < .01 are downregulated genes, and the genes with Log2FC > 0.5 and P-value < .01 are upregulated genes. (**D**) Real Time quantitative PCR (RT-qPCR) data showing relative *MDM2* gene expression levels assessed at 4 h (left panel) and 10 h (right panel) in untreated and QN-302 (0.4, 0.6, and 0.8 μM)-treated LPS cells (WD, DD-1, and DD-2). Results have been reported as relative quantity in treated versus untreated cells according to the 2^−ΔΔCt^ method. Data are reported as mean values ± SEM (*n* = 3). Statistical analyses were performed using the two-way ANOVA test by GraphPad Prism 10.0.3 (*P < .05, **P < .01, ***P < .001).

Considering the relevance of *MDM2* as a therapeutic target in LPS and our previous observation that a G-rich sequence within the *MDM2* P2 promoter, just upstream of the p53-binding site, can fold into very stable G4s (Fig. 2A) [19], we checked whether the *MDM2* promoter was G4-folded in live cells in our CUT&Tag dataset. We identified multiple G4 peaks within the region encompassing the 5′ untranslated region (5′ UTR) and first intron (P2 promoter) of the *MDM2* gene in LPS cells (Fig. 2B and Supplementary Fig. S1). Conversely, no G4s were detected at the same genomic site in the normal PA cells (Fig. 2B). The significant enrichment of G4s observed at the *MDM2* P2 promoter and the promising activity of QN-302 exerted in all tested LPS cells, prompted us to investigate whether QN-302 could directly interact with the *MDM2* G4s and hence directly affect *MDM2* gene expression levels. Having validated the *in vitro* binding and stabilization of MDM2-G4 by QN-302 by thermal unfolding CD spectroscopy analysis (Supplementary Fig. S2), CUT&Tag and RNA sequencing (RNA-seq) analysis was carried out in LPS and PA cells exposed for 4 h to a subtoxic amount of QN-302 (0.8 μM, i.e. <10% inhibition of cell viability). The correlation plot (Supplementary Fig. S3) and principal component analysis (Supplementary Fig. S4) relative to RNA-seq data indicated good grouping among the biological replicates. Like the CUT&Tag dataset obtained under no-drug conditions, the presence of QN-302 revealed multiple G4 peaks within the 5′ UTR and P2 promoter region of the *MDM2* gene in LPS cells (Fig. 2B), whereas no G4s were detected in normal PA cells. Interestingly, the intensity of the G4 peaks within the P2 promoter increased in all LPS cells after QN-302 treatment (Fig. 2B). In general, our findings indicate an increase of >4-fold in G4 levels (induced or stabilized *de novo*) across all QN-302-treated cell lines compared with their respective untreated controls (Supplementary Fig. S5). Notably, PA cells exhibited significantly fewer G4 peaks even after QN-302 treatment, confirming that normal cells have a markedly different G4 landscape compared with cancer cells [16]. Differential gene expression analysis performed on G4-enriched genes identified and selected by CUT&Tag revealed that over 60% of these genes are downregulated and contain a G4 element in their promoter in QN-302-treated LPS cells (Supplementary Fig. S6). These findings suggest a strong correlation among QN-302 treatment, G4 peaks, and gene transcription regulation. Notably, a significant downregulation of *MDM2* expression levels in QN-302-treated LPS cells compared with untreated cells has been observed (Fig. 2C). In contrast, no changes in the levels of *MDM2* transcript were detected in PA cells (Fig. 2C), which is in agreement with the CUT&Tag data showing that *MDM2* transcripts in PA cells are not included in those derived from G4-containing genes (Fig. 2B). Among the significantly downregulated G4-enriched genes upon QN-302 treatment (Supplementary Table S1) in common between LPS cells (WD, DD-1, and DD-2), *MDM2* was the only overexpressed gene, which incidentally is directly associated with the carcinogenesis of LPS. Given the pivotal role of *MDM2* in the pathogenesis of LPS, we focused our analysis on the interaction between QN-302 and the *MDM2* gene to explore the potential for a therapeutic window for LPS treatment.

To complement these data, we treated cells with subtoxic concentrations (0.4, 0.6, and 0.8 μM) of QN-302 for 4 and 10 h, as the expression of *MDM2* follows an oscillatory and cyclic pattern lasting ∼6 h [19]. After a 4-h treatment, a dose-dependent reduction of *MDM2* mRNA levels was observed in DD-2 cell lines and to a lesser extent in WD and DD-1 cells (Fig. 2D). Conversely, a 10-h exposure to QN-302 resulted in a remarkable increase of *MDM2* transcript levels in all LPS cells, especially at the lowest tested compound concentration (0.4 μM) (Fig. 2D). This evidence could indicate that at short timepoints (4 h), the interaction of QN-302 with the MDM2-G4 results in an inhibitory effect on *MDM2* transcription, which may be hindered by the complex autoregulatory feedback loop existing between MDM2 and p53 proteins [8] at greater timepoints (10 h).

### The p53-MDM2 autoregulatory feedback loop interferes with the inhibitory effect of QN-302 on *MDM2* transcription in LPS cells

**Figure 3.**
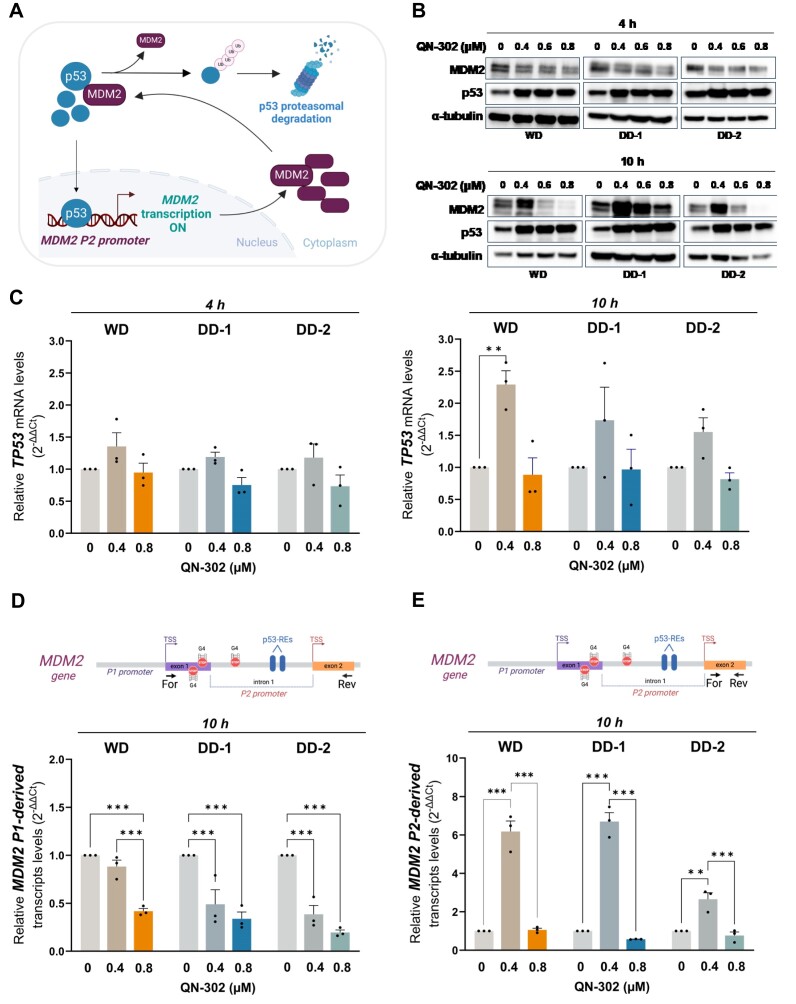
The inhibitory effect of QN-302 on *MDM2* increases p53 levels by virtue of the p53-MDM2 feedback loop. (**A**) Graphical representation of the p53-MDM2 autoregulatory feedback loop. Briefly, p53 enhances the expression of MDM2 protein by binding to specific REs within the P2 promoter. Conversely, MDM2 prevents its transcription by binding to p53 and targeting it to proteasome. Created in BioRender: Tosoni, B. (2025), https://BioRender.com/l36p271. (**B**) Representative immunoblotting showing the abundance of MDM2 and p53 proteins at 4 h (top panel) and 10 h (bottom panel) in untreated and QN-302-treated WD, DD-1, and DD-2 cells. α-tubulin was used to ensure equal protein loading. Cropped images of selected proteins have been shown. (**C**) Relative *TP53* gene expression levels assessed at 4 h (left panel) and 10 h (right panel) in untreated and QN-302-treated WD, DD-1, and DD-2 cells. Results have been reported as relative quantity in treated versus untreated cells according to the 2^−ΔΔCt^ method. Data represent mean values ± SEM (*n* = 3). Statistical analyses were performed using the two-way ANOVA test by GraphPad Prism 10.0.3 (*P < .05, **P < .01, ***P < .001). (D–E) RT-qPCR data showing the expression level of *MDM2* P1-derived (**D**) and P2-derived (**E**) transcript levels assessed at 10 h in untreated and QN-302-treated WD, DD-1, and DD-2 cells. Results are reported as relative quantity in treated versus untreated cells according to the 2^−ΔΔCt^ method. Data represent mean values ± SEM (*n* = 3). Statistical analyses were performed using the two-way ANOVA test by GraphPad Prism 10.0.3 (*P < .05, **P < .01, ***P < .001). A schematic representation of the primers pair design (For: forward; Rev: reverse) is reported at the top. Created in BioRender: Tosoni, B. (2025), https://BioRender.com/l36p271.

Considering that our LPS cell lines harbour a wild-type p53 and that MDM2 interacts with it and promotes the proteasomal degradation (i.e. reduced stability) of p53 protein, we investigated whether QN-302-mediated modulation of *MDM2* expression levels had an impact on p53 protein levels. To this end, we performed a KEGG enrichment pathway analysis focusing on the p53 signalling pathway. This analysis revealed that *MDM2* is consistently and significantly downregulated upon QN-302 treatment (0.8 μM) for 4 h across all three LPS cell lines, unlike other genes in the pathway, which exhibit variable upregulation or downregulation depending on the cell line (Supplementary Fig. S7). This observation suggests that *MDM2* is a consistent target of QN-302. Notably, alongside *MDM2*, another gene, *SESN2*, was significantly downregulated in all three LPS cell lines (Supplementary Fig. S7). However, unlike *MDM2*, *SESN2* was not highly expressed in these cell lines and in physiological conditions, a G4 structure was not present in the *SESN2* promoter in DD-1 and DD-2 cells (Supplementary Fig. S8). Conversely, a significant induction of a G4 peak at the *SESN2* promoter upon QN-302 (0.8 μM) treatment for 4 h was observed in the three LPS cell lines, but not in PA cells (Supplementary Fig. S8). This finding indicates that the downregulation of *SESN2*, like that of *MDM2*, is likely driven by the stabilization of G4 structures within the promoter region. In line with the observed decline in *MDM2* transcript levels (Fig. 2D, left panel), a reduction in MDM2 protein amounts was appreciable after 4 h treatment of LPS cells with different QN-302 concentrations (0.4, 0.6, and 0.8 μM) [Fig. 3B (top panel) and Supplementary Fig. S9]. Such an effect was paralleled by an increase of p53 protein [Fig. 3B (top panel) and Supplementary Fig. S9], suggesting that QN-302-mediated decrease of MDM2 protein at early timepoints could result in enhanced stability or reduced degradation of p53 protein. This conclusion gained further support by the observation that p53 mRNA levels were slightly affected at 4 h (Fig. 3C, left panel). However, while the same pattern of p53 protein accumulation was evident at 10-h exposure of WD and DDLPS cells to the same QN-302 concentrations (0.6 and 0.8 μM) that induced MDM2 protein downmodulation [Fig. 3B (bottom panel) and Supplementary Fig. S9], a marked accumulation of p53 was also observed in LPS cells exposed to 0.4 μM QN-302, the lowest concentration that elicited MDM2 accumulation at 10 h [Fig. 3B (bottom panel) and Supplementary Fig. S9]. The well-established complex autoregulatory feedback loop existing between MDM2 and p53, where the stress-mediated activation of p53 promotes the expression of *MDM2* from the inducible P2 promoter, which in turn targets p53 for proteasomal degradation, would help explain the counterintuitive results we obtained in terms of increased levels of both proteins at 10 h. A remarkable increase in the levels of p53 mRNA was indeed observed at 10 h and 0.4 μM of QN-302, (Fig. 3C, right panel). Altogether, these data suggest that at short timepoints the accumulation of p53 may be the result of its reduced degradation/increased stability consequent to QN-302-mediated reduction of MDM2 protein. Whereas at later timepoints, the exposure of LPS cells to QN-302 may result in a stress-induced accumulation of p53, which in turn masks the ligand-mediated inhibitory effect on *MDM2* and contributes to raising MDM2 protein levels by virtue of the autoregulatory feedback loop. To further assess the molecular mechanisms responsible for the results observed at 10 h, we dissected *MDM2* transcription using a specific primer pair to measure only the abundance of transcript derived from the P1 promoter (Fig. 3D, top panel) and P2 promoter (Fig. 3E, top panel). A 10-h exposure of LPS cells to QN-302 resulted in a dose-dependent reduction in the abundance of P1-derived *MDM2* transcript in all LPS cells (Fig. 3D, bottom panel). These data indicate that QN-302 interaction with the *MDM2* G4s within the 5′ UTR and P2 promoter regions, upstream of the p53-binding sites, inhibits RNA polymerase II (RNA Pol II) progression from the P1 promoter. The resulting low levels of MDM2 protein led to reduced degradation of p53, which in turn binds to the P2 promoter (Supplementary Fig. S10) and stimulates *MDM2* transcription (Fig. 3E, bottom panel). The highest QN-302 dosage in LPS cells significantly inhibited *MDM2* transcription by stabilizing G4s within the first exon and the intronic p53-inducible promoter (P2 promoter) of the *MDM2* gene. This stabilization may also hinder p53 binding to the P2 promoter of *MDM2* by altering the chromatin context. Evidence from CUT&Tag analysis following treatment revealed an increase in G4 peaks specifically within the p53-inducible promoter of the *MDM2* gene (Fig. 2B).

**Figure 4.**
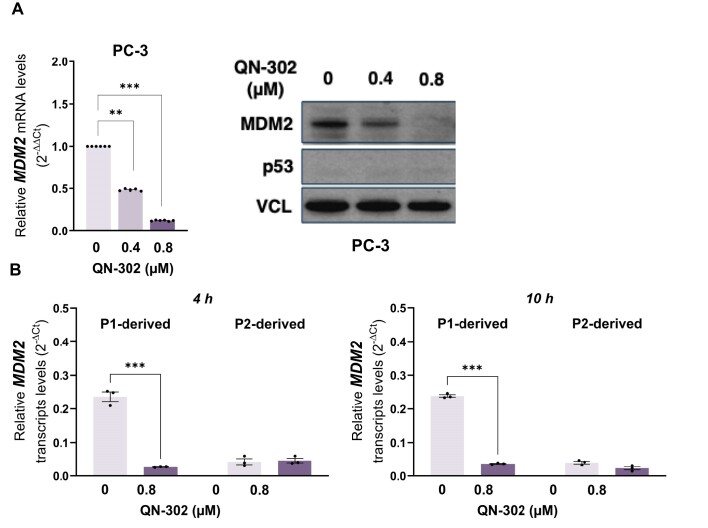
p53-induced *MDM2* P2 transcription triggered by QN-302 is abolished in a p53^null^ prostate cancer cell line. (**A**) Assessment of *MDM2* mRNA levels and protein abundance in p53^null^ PC-3 cells untreated and after 24-h exposure to QN-302 (0.4 and 0.8 μM). RT-qPCR data (left panel) are reported as relative quantity in treated versus untreated cells according to the 2^−ΔCt^ method and represent mean values ± SD. (**P< .01, ***P< .001; two-tailed Student’s *t*-test; *n* = 6). Representative western blotting (right panel) showing MDM2 protein abundance in untreated and QN-302-treated PC-3 cells. Cropped images of selected proteins are shown. Vinculin (VCL) was used to ensure equal protein loading. (**B**) RT-qPCR data showing the levels of nascent *MDM2* mRNA derived from P1 and P2 gene promoter in PC-3 cells after 4- and 10-h exposure to 0.8 μM QN-302. Data are reported as 2^−ΔCt^ and represent mean values ± SD (*n* = 3). Statistical analyses were performed using the two-way ANOVA test by GraphPad Prism 10.0.3 (***P < .001).

We next used p53^null^ prostate cancer PC-3 cells to verify whether increased transcription at the P2 promoter was dependent on p53. A remarkable downmodulation of *MDM2* mRNA and protein amounts was observed in p53^null^ PC-3 cells exposed to 0.4 and 0.8 μM of QN-302 (Fig. 4A), concentrations at which their cell growth was reduced by <50% (Supplementary Fig. S11). This evidence supports a direct role of p53 in the accumulation of MDM2 protein observed in LPS cells upon exposure to QN-302. The ability of QN-302 to interfere with *MDM2* transcription was further investigated by assessing the levels of newly synthesized (i.e. nascent) RNA transcripts originating from the *MDM2* P1 promoter. A marked decrease in the levels of P1-derived nascent RNA was observed in PC-3 cells exposed for 4 and 10 h to 0.8 μM QN-302, while no significant changes were observed in the nascent transcript levels from the P2 promoter (Fig. 4B). This evidence indicates that QN-302-mediated stabilization of the G4 within the P2 promoter likely directly interferes with *MDM2* gene transcription from the P1 promoter.

### QN-302 activates apoptosis

**Figure 5.**
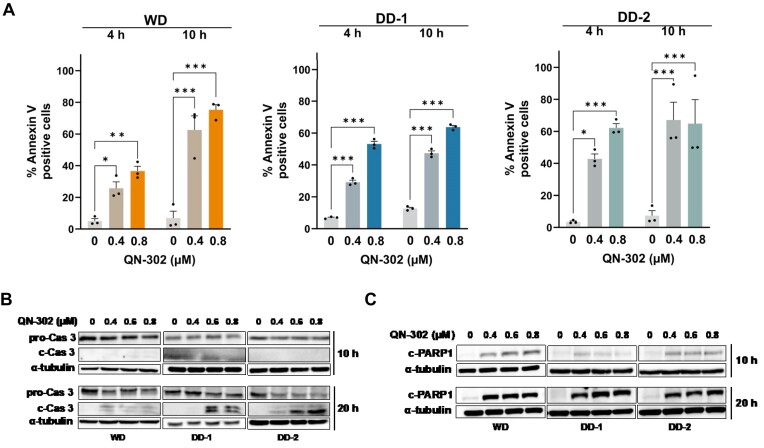
QN-302 induces activation of the apoptosis pathway in LPS cells. (**A**) Activation of early apoptotic signal assessed by Annexin V staining in WD, DD-1, and DD-2 cells after 4- and 10-h exposure to QN-302. Flow cytometry was used to capture 10 000 events per second and the percentage of Annexin V-positive cells was calculated for each condition. Data are reported as mean values ± SEM (*n* = 3). Statistical analysis was performed using the two-way ANOVA test by GraphPad Prism 10.0.3 (*P < .05, **P < .01, ***P < .001). (**B**) Representative western blot showing the abundance of pro-caspase 3 (pro-Cas 3), cleaved caspase 3 (c-Cas 3), and (**C**) cleaved PARP-1 (c-PARP1) proteins at 10 h (top panel) and 20 h (bottom panel) in untreated and QN-302-treated WD, DD-1, and DD-2 cells. Cropped images of the selected proteins are shown. α-tubulin was used to ensure equal protein loading.

To determine whether the high levels of p53 induced by QN-302 led to apoptotic cell death, we first performed Annexin V staining. Flow cytometric analyses revealed apoptosis activation in all QN-302-treated LPS cell lines starting at 4 h (Fig. 5A). We further observed the cleavage of the pro-enzyme form of caspase 3 into effector caspase 3, which reached a maximum after 20-h exposure to QN-302 (Fig. 5B). In all three different LPS cell lines, caspase 3 activation caused by QN-302 determined proteolytic degradation of PARP-1, indicating the activation of late apoptotic events. After 20 h, late apoptotic events, such as cleavage of the pro-enzyme form of caspase 3 into effector caspase 3, determined proteolytic degradation of PARP-1 (Fig. 5C). This phenomenon was observed in all WD/DDLPS cell lines (Fig. 5C).

### Harnessing the therapeutic potential of QN-302 in DDLPS cells

**Figure 6.**
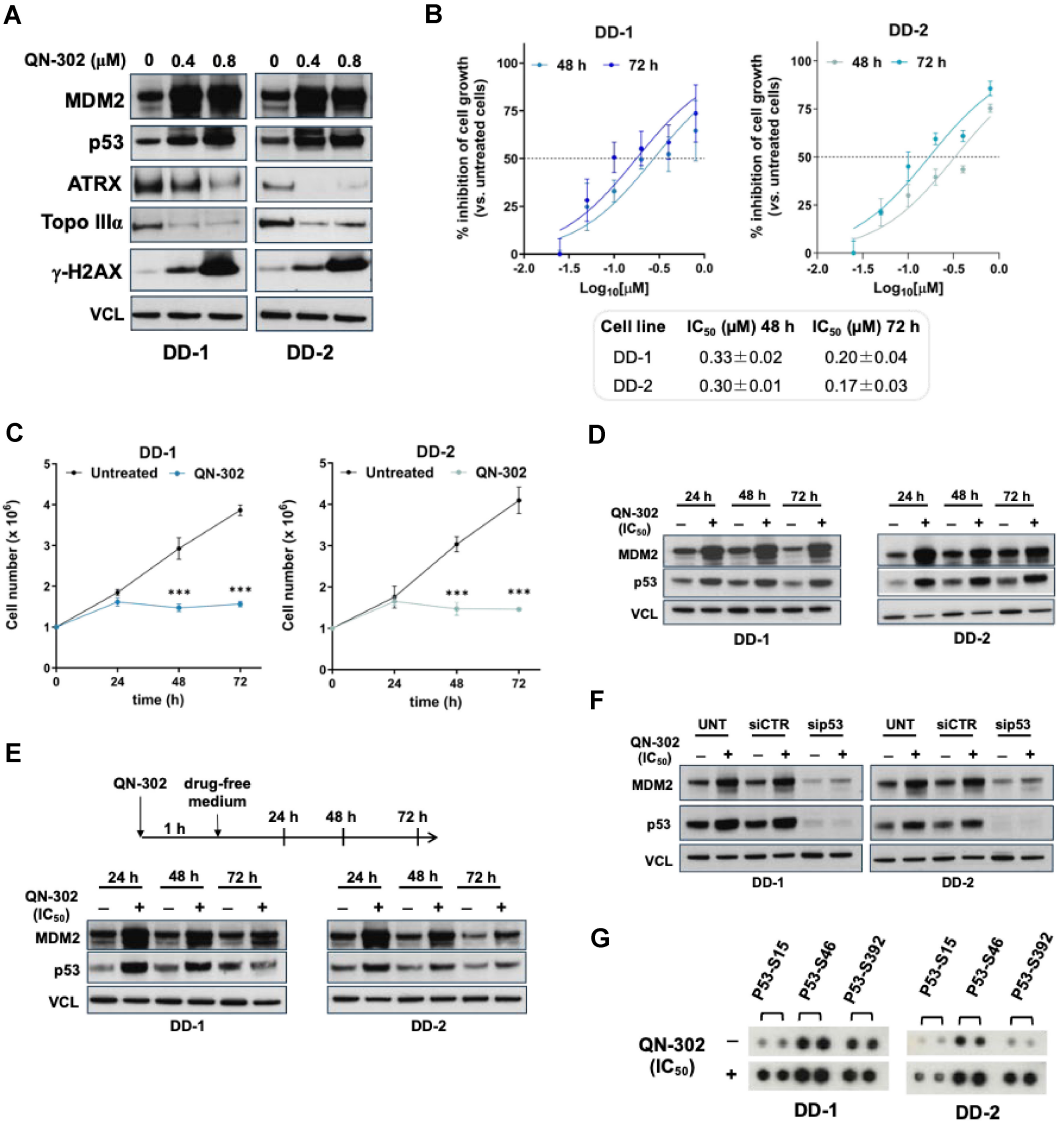
QN-302-mediated increase of p53 levels inhibits DDLPS cell growth. (**A**) Representative western immunoblotting showing the abundance of the indicated proteins in DDLPS cells untreated or exposed for 24 h to 0.4 and 0.8 μM QN-302. (**B**) Dose-response curves of DD-1 and DD-2 cells exposed for 48 and 72 h to increasing concentrations (from 0.025 to 0.8 μM) of QN-302. Data are reported as the percentage inhibition of cell growth with respect to untreated cells as a function of the Log_10_ compound concentrations and represent mean values ± SD from at least three independent experiments. (**C**) Time-dependent assessment of cell growth in DDLPS cells untreated or exposed to QN-302 (IC_50_ at 48 h). Data are reported as the number of growing cells over time and represent mean values ± SD (*n* = 5). ***P< .001 (two-tailed Student’s *t*-test; *n* = 5). (**D**) Representative western immunoblotting showing the abundance of MDM2 and p53 proteins at 24, 48, and 72 h in untreated (−) and QN-302 (IC_50_ at 48 h)-treated (+) DDLPS cells. (**E**) Representative western immunoblotting showing the abundance of MDM2 and p53 proteins in DDLPS cells, untreated (−) and after a 1-h exposure to QN-302 (0.8 μM), followed by the recovery for 24, 48, and 72 h in drug-free medium. The drawing on the top depicts the experimental scheme. (**F**) Representative western immunoblotting showing the abundance of MDM2 and p53 proteins in non-transfected (UNT), siCTR-, and sip53-transfected DD-1 and DD-2 cells either untreated (−) and after a 48-h exposure (+) to QN-302 (IC_50_ at 48 h). In all western immunoblotting data, cropped images of selected proteins are shown. VCL was used to ensure equal protein loading. (**G**) Representative blot from the Human Phospho-Kinase Proteome Profiler Array showing the phosphorylation status of p53 at serine S15, S46, and S392 in DDLPS untreated (−) and after a 48-h exposure to an equitoxic amount (IC_50_ at 48 h) of QN-302.

The therapeutic potential of QN-302 was further examined to assess if the anti-proliferative effects observed at 4, 10, and 20 h of treatment continued over time in our most aggressive patient-derived cell lines representing treatment-naïve, MDM2-positive, advanced (grade III) DDLPS. Specifically, the exposure of DD-1 and DD-2 cells to 0.4 and 0.8 μM QN-302 for 24 h was sufficient not only to favour MDM2 and p53 accumulation (Fig. 6A), but also to induce a dose-dependent reduction in the protein levels of ATRX and Topo IIIα (Fig. 6A), two factors known to protect cells from the replicative stress induced by small molecule stabilizers of G4s [42, 43]. These events were paralleled by the occurrence of DNA damage as suggested by the dose-dependent accumulation of γ-H2AX in both DDLPS cell lines (Fig. 6A). In addition, 48- and 72-h exposure of DDLPS cells to a narrow range of compound concentrations (from 0.025 to 0.8 μM) resulted in a dose-dependent inhibition of cell growth (Fig. 6B), with calculated IC_50_ values within the sub-micromolar range (Fig. 6B). The treatment of DD-1 and DD-2 cells with an equitoxic amount of QN-302 (IC_50_ at 48 h, Fig. 6B) resulted in a time-dependent inhibition of cell proliferation (Fig. 6C), which was paralleled by the accumulation of p53 and MDM2 protein amounts (Fig. 6D). This evidence is in line with the increase in the amounts of p53 in the presence of elevated levels of MDM2 protein observed in DDLPS cells exposed for 10 h to 0.4 μM of QN-302 (Fig. 3B) and further supports the pivotal role of p53 in stimulating the expression/accumulation of MDM2, according to the tumour promoting nature of the MDM2-p53 pathway in tumours characterized by *MDM2* amplification and bearing the wild-type form of p53. Consistent with this was the evidence that a 1-h pulse of 0.8 μM QN-302 in DDLPS cells resulted in the accumulation of both factors in cells recovered in drug-free medium for 24 h, followed by a progressive reduction in the levels of both proteins at subsequent timepoints (48 and 72 h; Fig. 6E). We confirmed by p53 silencing that the observed increase in MDM2 protein levels was dependent on the presence of a functional p53 protein. Indeed, RNA interference (RNAi)-mediated silencing of p53 resulted in the decrease of both basal and QN-302-induced MDM2 levels (Fig. 6F), even if a slight increase in MDM2 protein levels was still appreciable in p53-silenced cells exposed to QN-302, likely due to residual p53 protein amounts (Fig. 6F).

In further support of the ‘masking’ effect of wild-type p53 on the ligand-mediated inhibition of *MDM2* expression was the evidence that a 48-h exposure of DDLPS cells to an equitoxic amount of QN-302 (IC_50_ at 48 h) resulted in an increase in the phosphorylation of p53 at specific serine residues (S15, S46, and S392; Fig. 6G), which is triggered by diverse stress stimuli [7]. It has been indeed reported that under stress conditions, different post-translational modifications, including phosphorylation at specific serine residues, alter the interaction of p53 with MDM2 leading to increased p53 stability and favouring its nuclear translocation, the transcriptional activation of p53 target genes, and the initiation of tumour suppressive responses [7].

**Figure 7.**
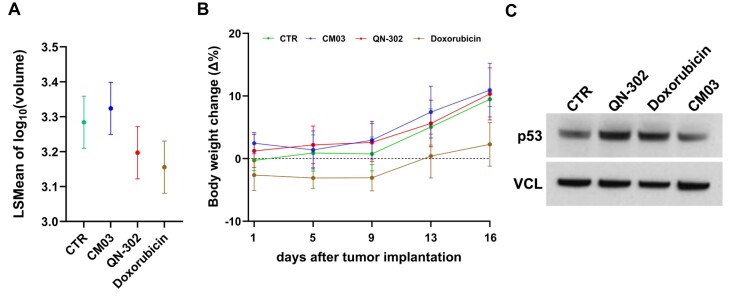
*In vivo* anti-tumour activity of QN-302 in a PDX model of DDLPS. (**A**) Distribution of TVs within control (CTR), CM03-, QN-302-, and doxorubicin-treated groups. Data are reported as the least square means of the log_10_ TV measured at the end of treatment (day 16) for each of the four groups. Dot points represent the least square mean, and the error bars indicate the corresponding 95% confidence intervals. (**B**) Time trend of cage-specific change of body weight according to treatment group. Data are reported as the percentage changes (Δ%) in body weight relative to the differences between times (days 1, 5, 9, 13, and 16 after tumour engraftment) in each cage according to treatment group: control, CM03, doxorubicin, and QN-302. (**C**) Representative western immunoblotting showing the levels of p53 protein in tumour specimens collected at the end of treatment from untreated (CTR), QN-302-, doxorubicin-, and CM03-treated mice. Cropped images of selected proteins are shown. VCL was used to ensure equal protein loading.

A previously established patient-derived xenograft (PDX) model, cognate to DD-2 cells and characterized by rapid *in vivo* growth (doubling time: 3.6 days; 95% C.I.: 2.9–4.7) [23] was initially used to explore the *in vivo* activity of QN-302, by applying dosage and treatment schedule previously described for the *in vivo* pancreatic ductal adenocarcinoma (PDAC) models [20, 40]. In line with our *in vitro* data, we observed that the distribution of TVs at the end of treatment (day 16) in QN-302-treated group was statistically different compared with that of CM03-treated group (*P*= 0.017) as well as that QN-302 exerted an anti-tumour activity that was comparable to that of the standard chemotherapeutic drug doxorubicin (*P*= 0.401) (Fig. 7A). Treatment schedules were well tolerated as no relevant body weight loss was appreciable over the time course of the experiment in treated versus control mice groups, except for a mild reduction (<10%) observed in the doxorubicin-treated group (Fig. 7B). Moreover, evidence of pharmacodynamic activity in terms of increased levels of p53 protein was observed for QN-302- and doxorubicin-treated groups with respect to untreated and CM03-treated mice (Fig. 7C).

## Discussion

The dismal prognosis and the low objective response rate to currently available therapeutic approaches represent a significant hurdle for the treatment of advanced/metastatic LPS [44]. This highlights the urgent need for the identification and validation of specific actionable targets for the development of novel precision medicine therapeutic approaches [6].

Compelling evidence has provided support for investigating MDM2 as a possible therapeutic target in WD/DDLPS with the final aim of reactivating the cellular intrinsic p53 tumour suppressor response [4, 7, 45]. Among the earliest MDM2-p53 antagonists, Nutlins (e.g. Nutlin 3a; idasanutlin RG7112) showed modest therapeutic efficacy and haematological toxicity in clinical trials [7]. Brigimadlin (BI907828; Boehringer Ingelheim) and milademetan (RAIN-32; RAIN Therapeutics), two MDM2 inhibitors in late developmental stages for LPSs, showed a manageable safety profile and encouraging signs of efficacy in *MDM2*-amplified WD/DDLPS patients [46]. However, no improvement in progression-free survival has been reported from a phase III study in advanced DDLPS patients treated with milademetan in comparison to trabectedin [7].

The stabilization of G4 structures by small molecules has emerged as an intriguing approach for the development of novel therapeutic strategies in cancer [17]. Several small molecules belonging to distinct chemical families have been indeed designed and characterized as G4 ligand during the last two decades. Some of them have shown significant anti-cancer activity, both as single agents and in combination with conventional anti-cancer drugs and radiation, in several pre-clinical models of human cancers [17].

The recent identification and characterization of a G4-forming sequence within the *MDM2* 5′ UTR and P2 promoter [19] has provided the rationale to explore this gene as a possible target amenable to small molecule-mediated G4 stabilization in LPS. In this study, we have reported the biological effects of the NDI-based G4 ligand QN-302 [18, 20, 21] in *MDM2*-amplified WDLPS cell line and patient-derived DDLPS pre-clinical models. Our data showed that QN-302 remarkably impairs the growth/viability of WDLPS and DDLPS cells in a dose-dependent manner, while only mildly affecting the growth/viability of normal PAs. Such an effect was associated with the abundance of cell-specific G4s, which were found to be predominantly located at promoter regions in cancer compared with normal cells. G4s within the region encompassing the *MDM2* 5′ UTR and p53-inducible P2 promoter, right upstream of the p53-binding sites, exclusively in WD/DDLPS and not in PA cells, represent excellent selective targets for this compound.

Here, we have shown that QN-302-mediated *MDM2* G4 stabilization hinders RNA Pol II progression from the constitutive P1 promoter in WD/DDLPS cells, thus inhibiting the formation of full-length *MDM2* transcript. In this context, a recent study showed that G4s downstream of the TSS have impact on transcription efficiency, with distinct effects based on their orientation: G4s in the template strand hinder RNA Pol II elongation, while G4s in the non-template strand influence transcription contingent on the chromatin environment. Strand displacement during transcription may favour both G4 folding and R-loop formation [47]. An elegant study revealed that the kinetics of G4s and R loops are not only highly similar in cells following treatment with G4 binders but also highly correlated with RNA Pol II stalling at those sites [14]. As the *MDM2* G4s reside in the template strand, we postulate that the G4/R loop interplay and G4 stabilization within the *MDM2* G4-positive region are responsible for hindering RNA Pol II elongation and hence the formation of the full-length P1-derived *MDM2* transcripts. Additionally, the reduction in the recruitment of transcription-binding factors and RNA Pol II at the promoter level due to G4 stabilization may also be accounted for. Indeed, it has been shown that G4 stabilization by small molecules globally decreases RNA Pol II occupancy at gene promoters in live cells. This stabilization modifies chromatin states hindering transcription initiation via inhibition of transcription factor recruitment at promoters [48].

QN-302-mediated downregulation of *MDM2* at early timepoints resulted in the deregulation of the p53-MDM2 autoregulatory feedback loop and, consequently, in the accumulation of p53. Consistent with this, the exposure of DDLPS cells to an equitoxic amount of the compound resulted in a time-dependent impairment of cell growth. Such an effect was associated with a persistent accumulation of p53, which was paralleled by a marked accumulation of MDM2 protein. This suggests that stress-induced accumulation and/or stabilization of p53 may contribute to raising MDM2 protein and P2-derived mRNA levels due to the autoregulatory feedback loop. It has indeed been reported that various forms of cellular stress, such as DNA damage, trigger p53 phosphorylation and hence its dissociation from MDM2, thus resulting in p53 protein stabilization and the subsequent upregulation of its downstream targets, including MDM2 itself [45]. In addition, QN-302-mediated stabilization of G4 may also induce replication-fork collapse because of obstruction of DNA polymerase progression, which generates DNA double-strand breaks and genome instability in cancer cells [14, 49]. Indeed, the exposure of DDLPS cells to QN-302 resulted in a dose-dependent reduction of factors (e.g. ATRX and Topo IIIα) known to protect cells from the replicative stress induced by chemical stabilizers of G4 structures, and in the occurrence of DNA damage. In this regard, activation of a DNA damage response could fuel p53 phosphorylation and consequently interfere with MDM2 interaction, thus favouring its further accumulation, eventually leading to apoptotic cell death. This evidence suggests that QN-302 represents a promising therapeutic tool to favour reactivation of p53 in tumours with wild-type *TP53* [45] and provides the rationale for further analyses, such as the design of drug combinations with standard chemotherapeutic drugs (i.e. doxorubicin) or other target-specific agents, such as CDK4 inhibitors [7] as well as *in vivo* treatment optimization (e.g. dose escalation, schedule, and route of administration) to be tested in additional DDLPS PDX models when available.

It would also be desirable in future studies, to access an inactive or scrambled analogue of QN-302, neither of which are currently available. However, in the absence of such compounds, the data for QN-302 compared with that for the earlier NDI derivative CM03 devised by us [40] do show a consistent trend in suggesting that differences in G4-binding ability correlate with differences in biological responses. Thus, (i) CM03 is consistently about 10-fold less potent in a panel of pancreatic cancer cell lines [20], (ii) it binds to G4s three- to five-fold less tightly [20], with reduced ability to stabilize G4s [18], and (iii) it is less effective in reducing LPS cell growth, showing IC_50_ values that are >3-fold higher than those of QN-302.

To date, few G4 ligands have entered early phases of clinical trials, though none of them has as yet progressed beyond phase II [17]. Quarfloxin (CX-3543), the first-in-class G4 ligand that reached phase II clinical trials for solid tumours, was withdrawn due to lack of efficacy as well as possible toxicity issues, whereas pidnarulex (CX-5461) is currently under evaluation in phase I clinical trial in patients with solid tumours bearing BRCA1/2, PALB2, or homologous recombination deficiency mutations and in patients with metastatic solid cancers (NCT04890613 and NCT06606990). Notably, QN-302 was granted orphan drug designation by the FDA at the beginning of 2023 (www.accessdata.fda.gov). It was subsequently granted clearance for a phase I, multi-centre, open-label, dose escalation and dose expansion trial for patients with advanced or metastatic solid tumours, including pancreatic ductal adenocarcinoma, prostate cancer, and sarcomas (NCT06086522). This trial started in November 2023 and is still on-going. Preliminary data from PDAC patients indicate that the drug is well tolerated at the low doses used to date, and that several instances of progression-free disease have been observed [50].

This study has demonstrated, to our knowledge for the first time, that targeting a single G4 may be therapeutically sufficient in those cancers that have a single (or a small number) of dominant aberrant driver genes especially in early-stage disease. On the other hand, complex hard-to-treat cancers involve dis-regulation of a large number of genes and their pathways, so that there are many G4s as potential targets. The exceptional G4 affinity and cellular potency of QN-302 may be important properties that enable it to have therapeutic potential in both categories, with PDAC and now LPS as current prime exemplars. We anticipate that a number of other cancers may be sensitive to QN-302.

Our data suggest that QN-302-mediated stabilization of G4s within the promoter of *MDM2* represents an intriguing approach to interfere with the MDM2-p53 pathway in WD/DDLPS for therapeutic purposes as well as in other *MDM2*-amplified cancers [46, 51, 52]. Moreover, the possibility that QN-302 acts at additional G4 targets other than *MDM2*, including telomeres or *CDK4*, may extend the therapeutic implications of the compound, an aspect worth of further investigation. Indeed, the G4 ligands reported thus far have shown a ‘multiple-target’ binding modality, which is likely an intrinsic feature that could be useful for the development of multi-hits, single-agent-based therapeutic approaches [53].

## Supplementary Material

gkaf085_Supplemental_Files

## Data Availability

CUT&Tag data were deposited into the Gene Expression Omnibus database under accession number GSE271822 and are available at the following URL: https://www.ncbi.nlm.nih.gov/geo/query/acc.cgi?acc=GSE271822. RNA-seq data were deposited into the Gene Expression Omnibus database under accession number GSE271823 and are available at the following URL: https://www.ncbi.nlm.nih.gov/geo/query/acc.cgi?acc=GSE271823.
