# Supplementary material for: The G-quadruplex experimental drug QN-302 impairs liposarcoma cell growth by inhibiting MDM2 expression and restoring p53 levels
Source: Nucleic Acids Res. 2025 Feb 13;53(4):gkaf085. doi: 10.1093/nar/gkaf085 (PMC11822379; doi:10.1093/nar/gkaf085)
Supplement: gkaf085_Supplemental_Files [file gkaf085_supplemental_files.zip › Tosoni et al. revised Supplementary Information.pdf]

Supplementary Information

The G-quadruplex experimental drug QN-302 impairs liposarcoma cell growth by inhibiting *MDM2* expression and restoring p53 levels

Beatrice Tosoni,[1†] Eisa Naghshineh,[2†] Irene Zanin,[1] Irene Gallina,[1] Lorenzo Di Pietro,[2] Loredana Cleris,[2] Matteo Nadai,[1] Mara Lecchi,[3] Paolo Verderio,[3] Pietro Pratesi,[3] Sandro Pasquali,[2] Nadia Zaffaroni,[2] Stephen Neidle,[4] Marco Folini,[2‡] and Sara N. Richter[1,5‡*]

[1]Department of Molecular Medicine, University of Padua, via A. Gabelli 63, 35121 Padua, Italy.

[2]Molecular Pharmacology Unit, Department of Experimental Oncology, Fondazione IRCCS Istituto Nazionale dei Tumori di Milano, Via G. A. Amadeo, 42, 20133 Milan, Italy

[3]Bioinformatic and Biostatistics Unit, Department of Epidemiology and Data Science, Fondazione IRCCS Istituto Nazionale dei Tumori di Milano, Via G. Venezian, 1, 20133 Milan, Italy;

[4]School of Pharmacy, University College London, London WC2N 1AX, United Kingdom

[5]Microbiology and Virology Unit, Padua University Hospital, Padua 35128, Italy

†Equally contributed

‡The authors share co-last authorship

* To whom correspondence should be addressed. Tel: +39 048272346; Email: sara.richter@unipd.it

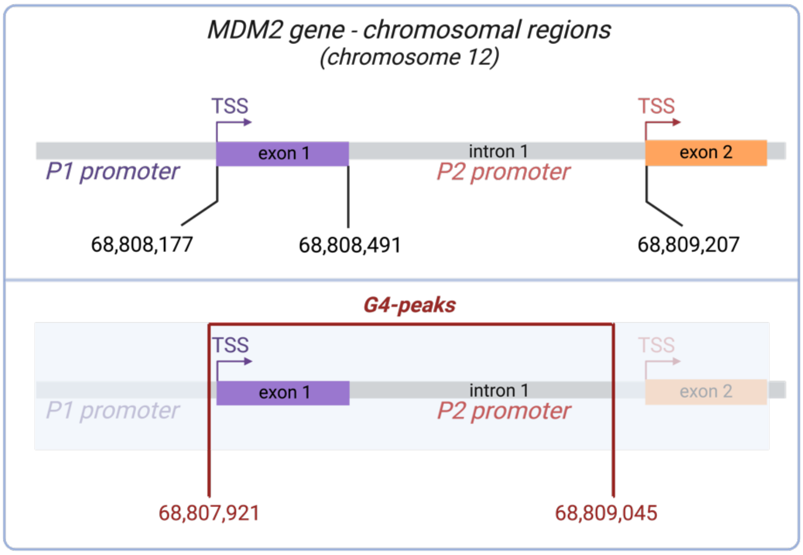

Supplementary Figure 1. Schematic representation of G4-peaks localization detected by G4 CUT&Tag within *MDM2* promoter in LPS cells. Chromosomal coordinates are reported.

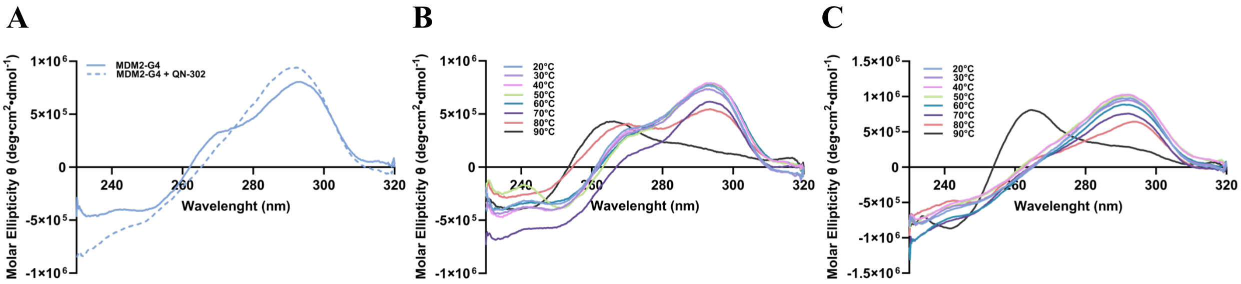

Supplementary Figure 2. **A**) CD spectra of MDM2-G4 in the absence and presence of 8 μM QN-302 in 25 mM KCl measured at 35°C. Thermal unfolding CD spectra of MDM2-G4 in the **B**) absence and **C**) presence of 8 μM QN-302, in a 25 mM KCl buffered solution. Being MDM2-G4 T_m above 90°C, we could not estimate its differential T_m in the presence and absence of the compound; however, stabilization is appreciable by CD spectra modification, especially at 70-90°C, in the presence of QN-302.

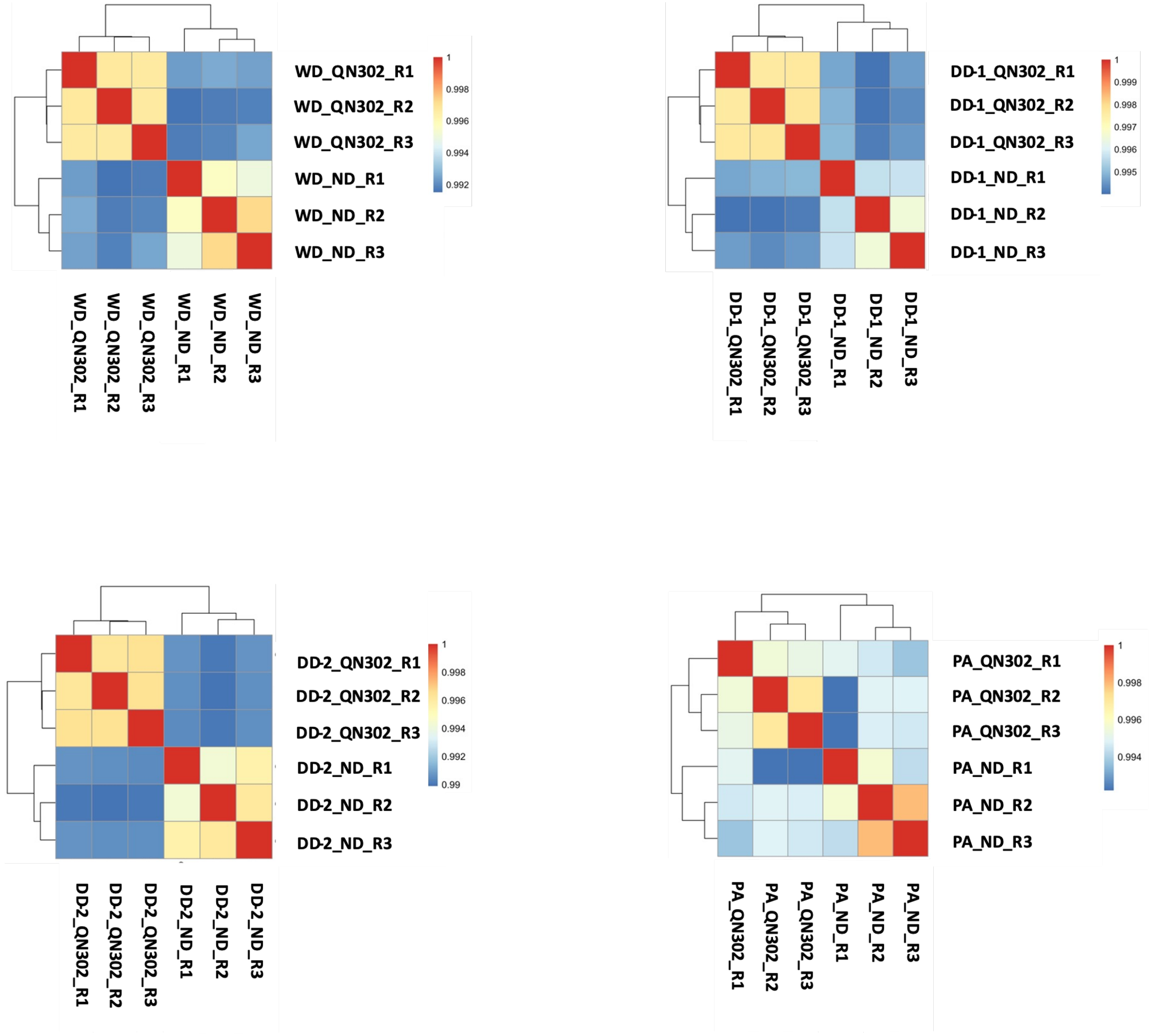

Supplementary Figure 3. Heatmap of the Pearson Correlation coefficient among RNA-seq data biological replicates (R) of WD, DD-1, DD-2 and PA cells in untreated (ND) and QN-302-treated (QN302) condition. Each coefficient in the correlation matrix is represented as a square which colour represents the level of correlation. The more similar two samples are, the more the correlation coefficient is equal to 1.

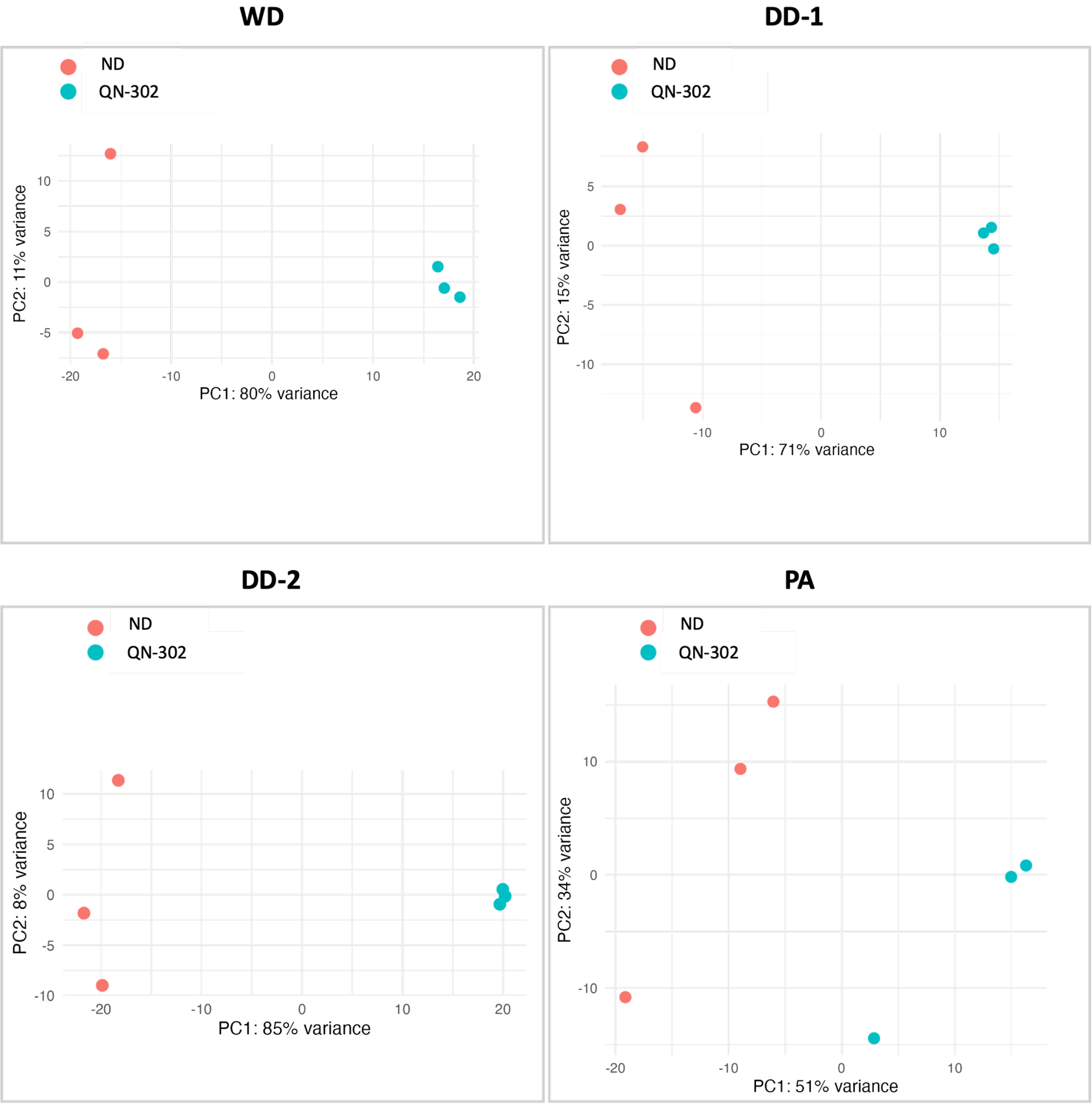

Supplementary Figure 4. Principle component analysis (PCA) and sample clustering among RNA-seq data biological replicates of WD, DD-1, DD-2 and PA cells in untreated (red dot) and QN-302-treated (blue dot) condition. PCA is a useful method to determine the variance within and across different experimental conditions and replicates. High variance is observed between the two experimental conditions (ND vs QN-302 treated), while a low variance value is observed among replicates within each condition.

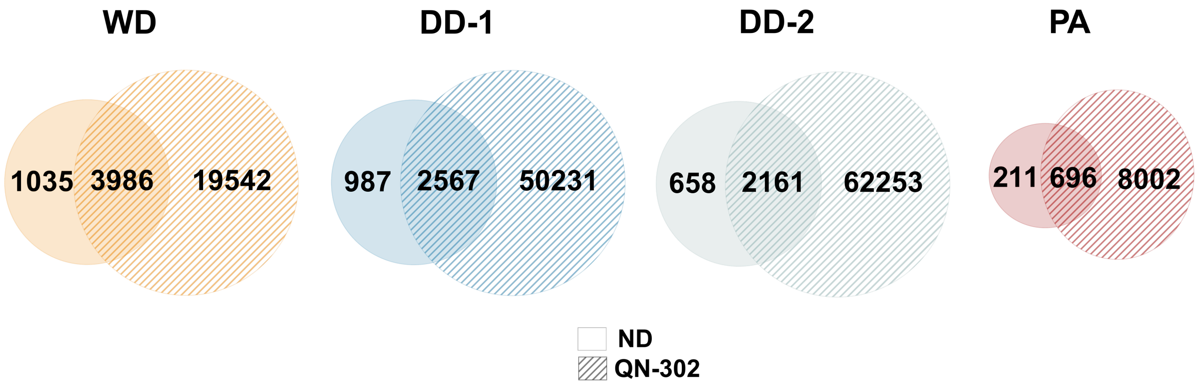

Supplementary Figure 5. Overlap of high confidence G4 peaks between untreated (**ND**) and QN-302-treated (**QN-302**) samples in each LPS (WD, DD-1 and DD-2) and PA cells obtained by G4 CUT&Tag. The two experimental conditions were conducted in parallel upon 4-h exposure to 0.8 µM QN-302 (**QN-302**) or not QN-302 (**ND**) for each cell line tested. High-confidence peaks were defined as those that were consistently detected in at least two out of three independent biological replicates.

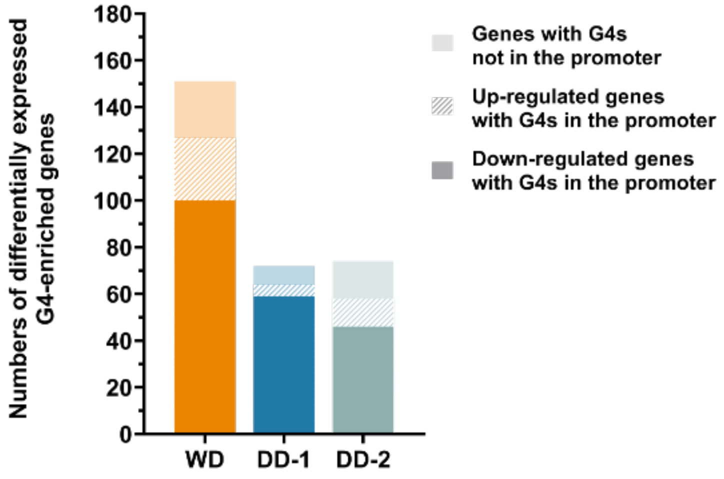

Supplementary Figure 6. Evaluation of the amount of the differentially expressed G4-enriched genes in LPS cells. For each cell line, the number of genes with and without G4s in their promoter is reported.

WD

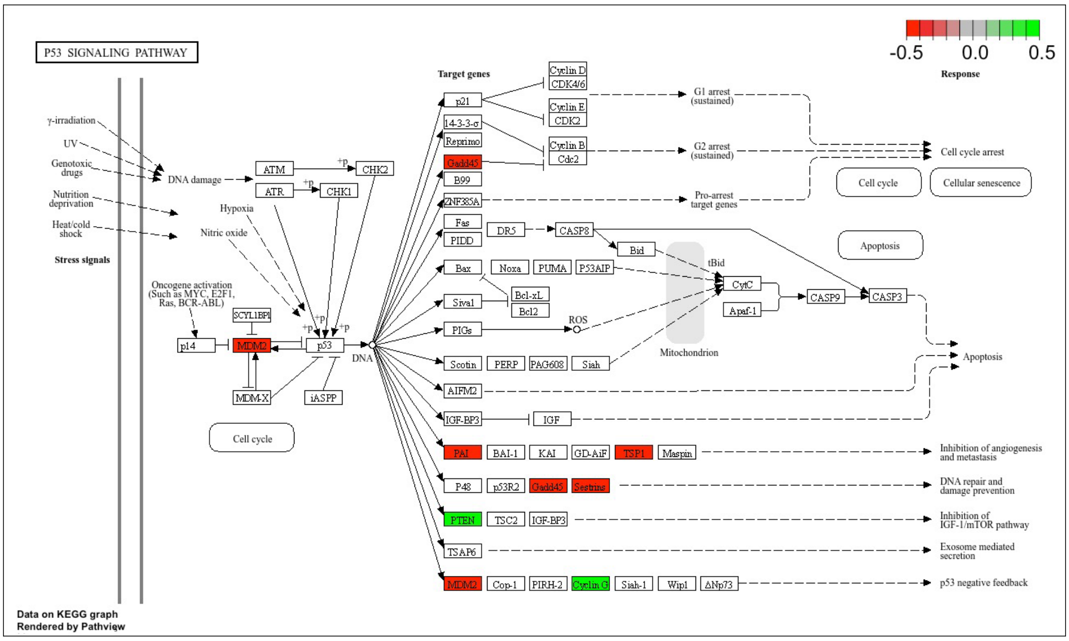
P53 SIGNALING PATHWAY
-0.5 0.0 0.5
Response
Target genes
γ-irradiation
UV
Genotoxic drugs
Nutrition deprivation
Heat/cold shock
Stress signals
DNA damage
ATM
ATR
CHK2
CHK1
Hypoxia
Nitric oxide
Oncogene activation (Such as MYC, E2F1, Ras, BCR-ABL)
SCYL1BP1
p14
MDM2
p53
MDM-X
iASPP
DNA
Cell cycle
p21
14-3-3-σ
Reprimo
Gadd45
B99
ZNF385A
Fas
PIDD
Bax
Sival
PIGs
Scotin
AIFM2
IGF-BP3
PAI
P48
PTEN
TSAP6
MDM2
Cyclin D
CDK4/6
Cyclin E
CDK2
Cyclin B
Cdc2
G1 arrest (sustained)
G2 arrest (sustained)
Pro-arrest target genes
Cell cycle arrest
Cell cycle
Cellular senescence
DR5
CASP8
Bid
tBid
Noxa
PUMA
P53AIP
Bcl-xL
Bcl2
ROS
CytC
Apaf-1
CASP9
CASP3
Mitochondrion
Apoptosis
PERP
PAG608
Siah
IGF
BAI-1
KAI
GD-AiF
TSP1
Maspin
p53R2
Sestrins
TSC2
Cop-1
PIRH-2
Cyclin G
Siah-1
Wip1
ΔNp73
Inhibition of angiogenesis and metastasis
DNA repair and damage prevention
Inhibition of IGF-1/mTOR pathway
Exosome mediated secretion
p53 negative feedback
Data on KEGG graph
Rendered by Pathview

DD-1

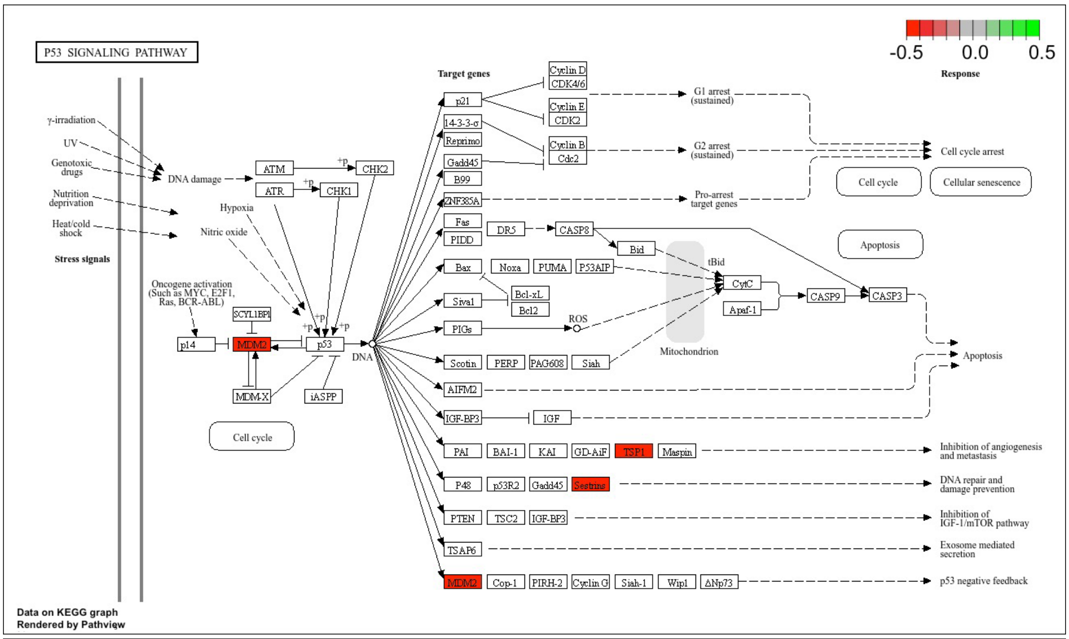
P53 SIGNALING PATHWAY
-0.5 0.0 0.5
Response
Data on KEGG graph
Rendered by Pathview

DD-2

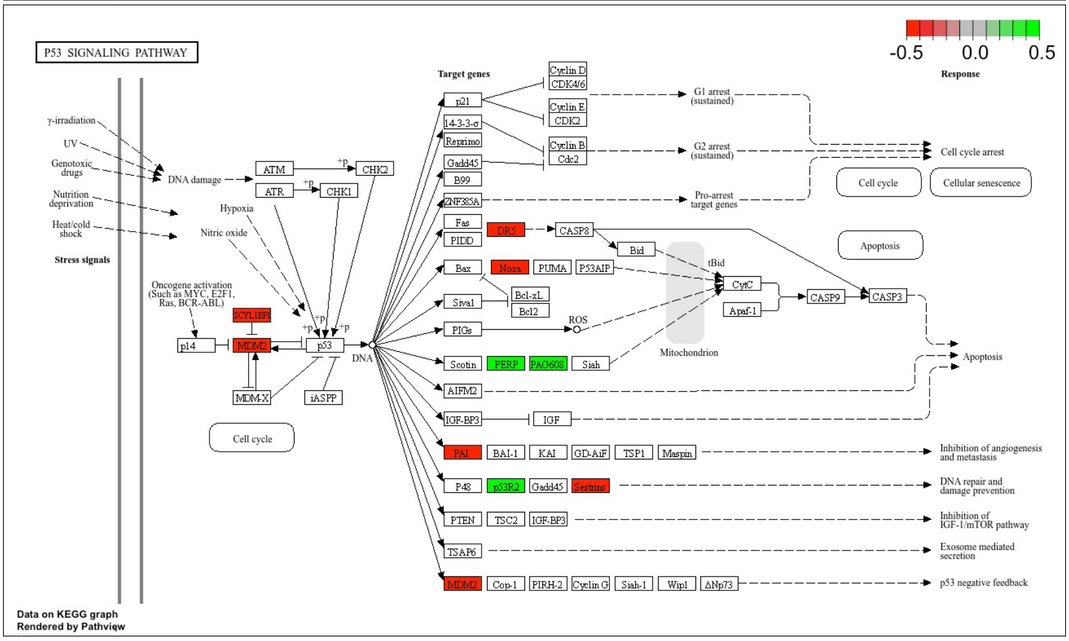
P53 SIGNALING PATHWAY
-0.5 0.0 0.5
Response
Data on KEGG graph
Rendered by Pathview

Supplementary Figure 7. Enrichment analysis of the p53 signaling pathway was conducted upon 4 h of QN-302 treatment (0.8 µM) in LPS cells (WD, DD-1, and DD-2) using RNA-seq experiments. KEGG pathway enrichment analysis was performed on the complete dataset of significant differentially expressed genes (DEGs). Significant DEGs are represented as follows: red for down-regulated genes (Log2FC < −0.5 and padj < 0.05) and green for up-regulated genes (Log2FC > 0.5 and padj < 0.05).

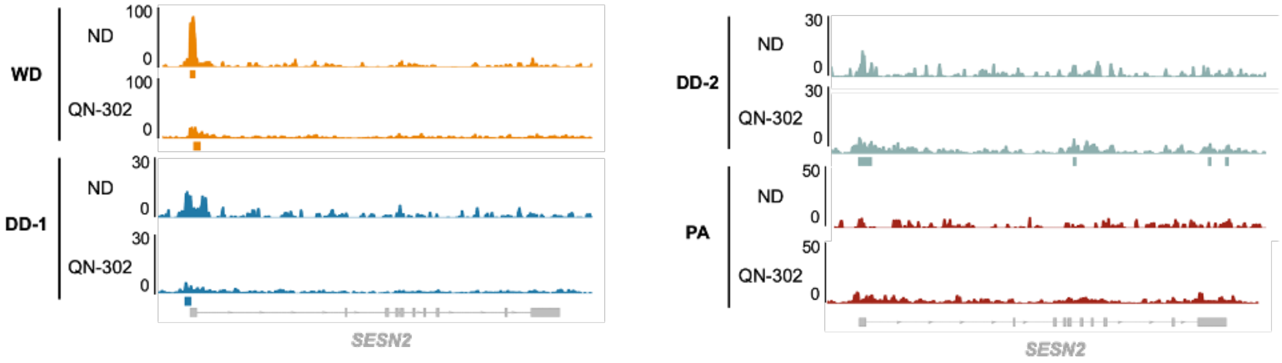

Supplementary Figure 8. Visualization of G4-CUT&Tag profiles of LPS and PA cells treated (QN-302) or untreated (ND) with QN-302 within the *SESN2* genomic region. Reads were aligned to the human genome (hg38) and normalized as RPGC. For each profile, MACS2 called peaks are reported as colored boxes under each track. Gene annotation is reported at the bottom of the figure.

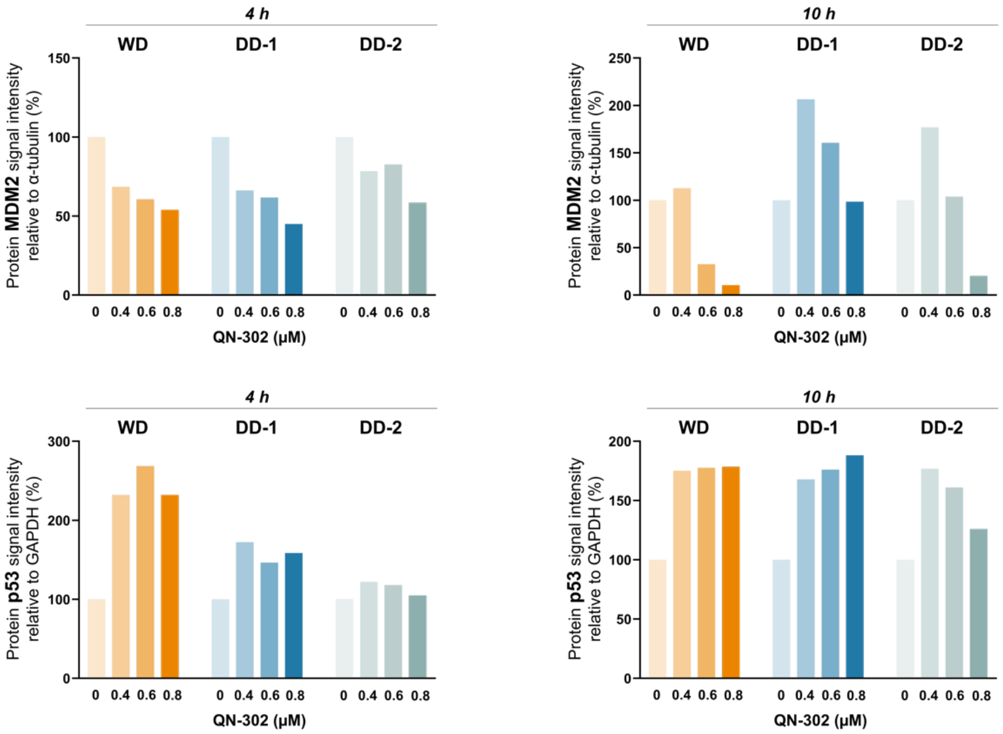

Supplementary Figure 9. Gel bands quantification of western blots analysis for MDM2 and p53 proteins shown in Figure 3. MDM2 protein signal intensity (%) was normalized to α-tubulin protein signal, while p53 protein signal intensity (%) was relative to GAPDH protein signal.

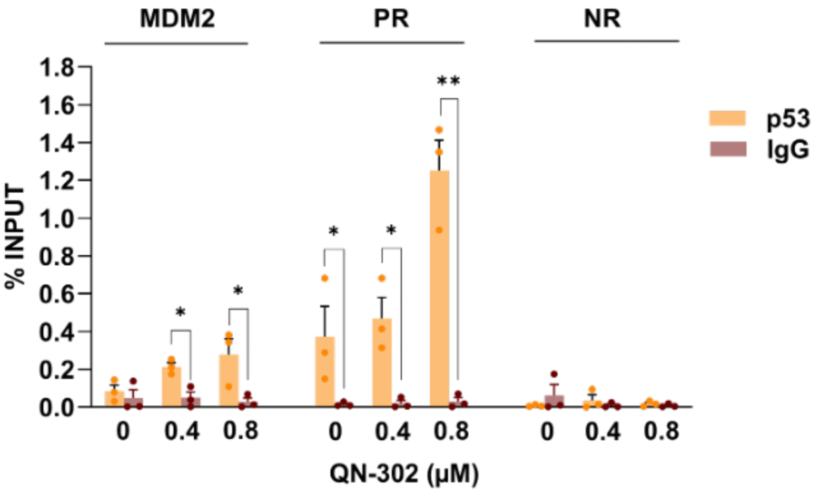

Supplementary Figure 10. ChIP-qPCR analyses of p53 binding at the *MDM2* P2 promoter region in WD cells untreated and treated with two different QN-302 concentrations (0.4 μM and 0.8 μM) for 4 h, compared to the negative control IgG. ChIP-qPCR amplicons were designed to amplify the following regions: MDM2 = target region, which is the p53-responsive element region located within the *MDM2* P2 promoter; PR = positive region, which is the p53-responsive element region located within the CDKN1A gene; NR = negative region, which is located almost 20 kb pairs downstream of the *MDM2* P2 promoter and does not contain p53-binding sites. Data shown are reported as relative to input and represent the mean ± SEM of three independent experiments. Statistical analyses were performed using two-tailed paired Student's t test. P-values are represented as: ∗P < 0.05 and ∗∗P < 0.01.

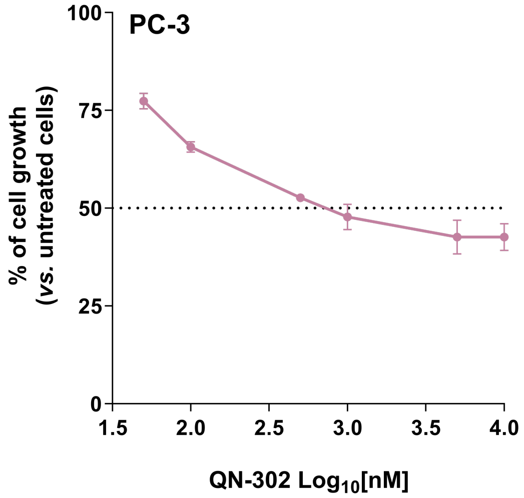

Supplementary Figure 11. Dose-response curves of PC-3 cells exposed for 24 h to increasing concentrations (from 0.05 to 10 μM) of QN-302. Data are reported as the percentage of cell growth with respect to untreated cells as a function of the Log10 compound concentrations and represent mean values ± s.d. from at least three independent experiments.

**Gene symbol**	**Gene name**	**G4-peak annotation**
MDM2	mouse double minute 2	Promoter (<=1kb)
EXOC8	exocyst complex component 8	Promoter (<=1kb)
MYH9	myosin heavy chain 9	Promoter (<=1kb)
PARS2	prolyl-tRNA synthetase 2, mitochondrial	Promoter (<=1kb)
PHF13	PHD finger protein 13	Promoter (<=1kb)
TP53RK	TP53 regulating kinase	Promoter (<=1kb)
VCPIP1	valosin containing protein interacting protein 1	Promoter (<=1kb)
ZBED4	zinc finger BED-type containing 4	Promoter (<=1kb)
ZBTB2	zinc finger and BTB domain containing 2	Promoter (<=1kb)
HAPSTR1	HUWE1 associated protein modifying stress responses	Distal intergenic
IFFO2	intermediate filament family orphan 2	Distal intergenic
ZNF212	zinc finger protein 212	Distal intergenic

Supplementary Table 1. List of the G4-enriched genes significantly down-regulated upon QN-302 treatment and shared among the three LPS cell lines (WD, DD-1 and DD-2).
